# Analyzing the Effects of Capacitances-to-Shield in Sample Probes on AC Quantized Hall Resistance Measurements

**DOI:** 10.6028/jres.104.023

**Published:** 1999-08-01

**Authors:** M. E. Cage, A. Jeffery

**Affiliations:** National Institute of Standards and Technology, Gaithersburg, MD 20899-0001

**Keywords:** ac quantum Hall effect, capacitance-to-shield, equivalent electrical circuit, longitudinal resistance, multi-series connections, quantized Hall resistance

## Abstract

We analyze the effects of the large capacitances-to-shields existing in all sample probes on measurements of the ac quantized Hall resistance *R*_H_. The object of this analysis is to investigate how these capacitances affect the observed frequency dependence of *R*_H_. Our goal is to see if there is some way to eliminate or minimize this significant frequency dependence, and thereby realize an intrinsic ac quantized Hall resistance standard. Equivalent electrical circuits are used in this analysis, with circuit components consisting of: capacitances and leakage resistances to the sample probe shields; inductances and resistances of the sample probe leads; quantized Hall resistances, longitudinal resistances, and voltage generators within the quantum Hall effect device; and multiple connections to the device. We derive exact algebraic equations for the measured *R*_H_ values expressed in terms of the circuit components. Only two circuits (with single-series “offset” and quadruple-series connections) appear to meet our desired goals of measuring both *R*_H_ and the longitudinal resistance *R_x_* in the same cool-down for both ac and dc currents with a one-standard-deviation uncertainty of 10^−8^ *R*_H_ or less. These two circuits will be further considered in a future paper in which the effects of wire-to-wire capacitances are also included in the analysis.

## 1. Introduction

Many laboratories are now attempting to employ the integer quantum Hall effect (QHE) [1–3] to realize an intrinsic ac resistance standard by using ac bridges to compare ac quantized Hall resistances *R*_H_ with ac reference standards. In experiments reported to date [4–9], the measured values of the ac quantized Hall resistances *R*_H_ unfortunately varied with the applied frequency *f* of the current, and differed from the dc value of *R*_H_ by at least 10^−7^ *R*_H_ at a frequency *f* of 1592 Hz (where the angular frequency *ω*= 2π*f* is 10^4^ rad/s). Furthermore, some sample probe leads had to be removed at the device in order to reduce the frequency dependence to this still significant amount. Lead removal creates two problems: (1) parasitic impedances within the QHE resistance standard (which arise from capacitances, inductances, lead resistances, and leakage resistances) become more difficult to measure or estimate, making it harder to apply corrections to the measured values of *R*_H_; and (2) measurements of both *R*_H_ and the longitudinal resistance *R_x_* can not be made during the same cool-down, which has been found to be necessary [10] in order to obtain reliable values of *R*_H_ with direct (dc) currents.

Our desired goal at NIST is to measure both *R*_H_ and the longitudinal resistance *R_x_* in the same cool-down for both ac and dc currents with all sample probe leads attached, and to do this with a one-standard-deviation uncertainty equal to or less than 10^−8^ *R*_H_ in order to verify and replace parts of the calculable capacitor chain [11] that provides the System International (SI) value of *R*_H_ at NIST. The one-standard-deviation uncertainty of the entire NIST calculable capacitor chain is 2.4 × 10^−8^ *R*_H_. Therefore, we need to achieve uncertainties of 10^−8^ *R*_H_ or less in the ac *R*_H_ measurements.

Therefore, the frequency dependence of *R*_H_ is a serious problem that must be addressed. This paper investigates the effects of the capacitances-to-shield, and the series inductances and series resistances of sample probe leads on measurements of the ac *R*_H_. It also identifies ways to eliminate or minimize the frequency dependences resulting from these parasitic impedances. Most of the capacitances-to-shield arise from the capacitances between the inner and outer conductors of the coaxial leads and connectors within the ac quantized Hall resistance standard; a smaller amount arises from the capacitances between the quantum Hall effect device plus sample holder and the surrounding conducting surfaces of the sample probe.

## 2. Strategy

We investigate the effects of capacitances-to-shield on measurements of *R*_H_ by using equivalent electrical circuits and multiple connections to the quantum Hall effect device. The multiple connections will be defined in Secs. 7–9. We derive exact algebraic equations for the currents and quantum Hall voltages of the standard. The discrete circuit components consist of: (a) capacitances and leakage resistances to the shields of the ac quantized Hall resistance standard; (b) inductances and series resistances of the internal and external sample probe leads and connectors; and (c) quantized Hall resistances, longitudinal resistances, and voltage generators within the quantum Hall effect device itself. These circuit components include everything within the standard except wire-to-wire capacitances between pairs of the inner conductors. Significant wire-to-wire capacitances can exist between pairs of conducting surfaces of the quantum Hall effect device, the sample holder, and the bonding wires between them. The wire-to-wire capacitances may be important, but their inclusion makes the circuit analyses extremely difficult, so they are excluded at this intermediate stage where we are trying to find viable circuit candidates for the final analysis of a complete equivalent circuit representation of an ac quantized Hall resistance standard.

We give a brief explanation of the dc quantum Hall effect in Sec. 3. Section 4 describes our equivalent electrical circuit model of an ac quantized Hall resistance standard. Single-series “normal”, single-series “offset”, double-series, and quadruple-series circuits are explained and analyzed in Secs. 5–7 and Sec. 9. We find that two of these circuits (those with single-series “offset” and quadruple-series connections) appear to meet our desired goals of measuring both *R*_H_ and the longitudinal resistance *R_x_* in the same cool-down for both ac and dc currents with an uncertainty of 10^−8^ *R*_H_ or less. These two circuits will be analyzed in more detail in a future paper in which the effects of wire-to-wire capacitances are also included in the analysis.

## 3. DC Quantum Hall Effect

The quantum Hall effect (QHE) has been successfully used as an intrinsic dc resistance standard. In the integer dc QHE [1–3], the Hall resistance *R*_H_ of the *i*th plateau of a fully-quantized, two-dimensional electron gas (2DEG) is *R*_H_(*i*) = *V*_H_(*i*)/*I*_T_, where *V*_H_(*i*) is the quantum Hall voltage measured between potential probes located on opposite sides of the device, and *I*_T_ is the total current flowing between the source and drain current contacts at the ends of the device. Under ideal conditions, the values of *R*_H_(*i*) in standards-quality devices satisfy the relationships *R*_H_(*i*) = *h*/(*e*^2^*i*) = *R*_K_/*i*, where *h* is the Planck constant, *e* is the elementary charge, *i* is an integer, and *R*_K_ is the von Klitzing constant, *R*_K_ ≈ 25 812.807 Ω [12]. However, the conditions are not always ideal. The values of *R*_H_(*i*) can vary with the device temperature *T* [13] and with the applied current *I*_T_ [14]. Thus the measured dc values of *R*_H_(*i*) are not necessarily equal to *h*/(*e*^2^*i*).

The current flow within the 2DEG is nearly dissipationless in the quantum Hall plateau regions of high-quality devices, and the longitudinal resistances *R_x_*(*i*) of this standard become very small over ranges of magnetic field in which quantized Hall resistance plateaus are observed. The dc longitudinal resistance is defined to be *R_x_*(*i*) = *V_x_*(*i*)/*I*_T_, where *V_x_*(*i*) is the measured longitudinal voltage drop between potential probes located on the same side of the device. The dc values of *R_x_*(*i*) can also be temperature [13] and current [14] dependent.

## 4. Equivalent Electrical Circuit of an AC QHE Standard

The quantized Hall resistance *R*_H_(*i*) of an ac QHE resistance standard (ac QHRS) can be experimentally compared with the impedances of ac reference standards using ac measurement systems. NIST initially plans to use ac resistors as reference standards, and an ac ratio bridge measurement system for the comparisons.

**Fig. 1:**
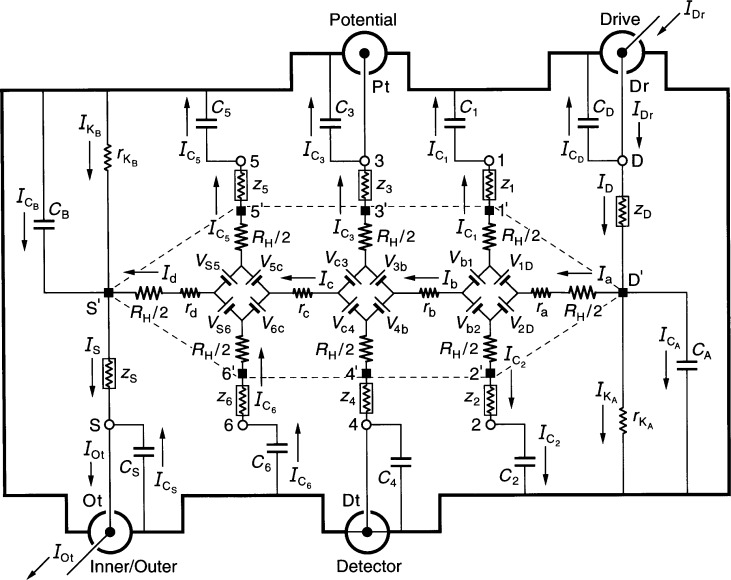
An equivalent electrical circuit representation of an ac QHE resistance standard with single-series “normal” connections to the device. The symbols are explained in Sec. 4. See Sec. 5 for the circuit analysis. The QHE device and the sample holder are located within the dashed-line region. The quantized Hall resistance is being measured in an ac ratio bridge using four-terminal-pair [15,16] measurement techniques. The ac ratio bridge is not shown, nor is the ac reference resistance standard with which the QHE standard is being compared.

Figure 1 shows an equivalent electrical circuit representation of an ac QHRS in which the QHRS is being measured with an ac bridge using four-terminal-pair [15,16] techniques. (Neither the ac reference standard nor the ac ratio bridge are shown in the figure.) This circuit of an ac QHRS is rather detailed, so we explain it one step at a time, starting with the periphery of the standard, then proceeding to the QHE device within the central region of the figure, and finally discussing properties of the sample probe leads within the standard.

The ac QHRS of Fig. 1 is bounded by an electrical shield indicated schematically by thick lines. Actual shields have complicated surface geometries. They consist of: (a) conductive surfaces surrounding the QHE device and its sample holder at liquid helium temperatures; (b) the outer conductors of eight coaxial leads within the sample probe; and (c) the outer conductors of eight coaxial leads extending from the top of the sample probe to room temperature access points S, 1 through 6, and D. The electrical shields will also be referred to in the text as “outer conductors”. To simplify the figure, we label only currents in the inner conductors.

The ac QHRS has electrical access at room temperature via four coaxial measurement ports labeled Inner/Outer, Detector, Potential, and Drive. These four ports are used in the four-terminal-pair measurements, where each coaxial port is referred to as a “terminal-pair”. The four coaxial ports are connected to room temperature access points S, 4, 3, and D in the figure.

The ideal four-terminal-pair measurement definition [15,16] of *R*_H_(*i*) is satisfied by the following three simultaneous conditions: (1) the current *I*_Dr_ at the Drive coaxial port is adjusted so that there are no currents in the inner or outer conductors of the Potential coaxial port, i.e., *I*_Pt_ = 0; (2) the potential difference is zero across the inner and outer conductors of the Detector coaxial port; and (3) there are no currents in the inner or outer conductors of the Detector coaxial port, i.e., *I*_Dt_ = 0.

It is implicit in the four-terminal-pair definition that each coaxial port is treated as a terminal-pair, and that the current in the inner conductor of every port is equal and opposite to the current in the outer conductor (the shield). Coaxial chokes [17] (located outside the ac quantized Hall resistance standard and therefore not shown in the figure) assure that this equal and opposite current condition is satisfied for each of the four terminal-pairs in the circuit. The current *I*_Ot_ exits the ac QHRS at the Inner/Outer port and enters the ac reference standard (not shown).

A “virtual” short has been drawn in Fig. 1 as a line between the shield and inner conductor at the Detector coaxial port to indicate four-terminal-pair condition number (2). We let the Detector potential be zero, i.e., *V*_Dt_ = 0. At bridge balance the ac quantized Hall voltage *V*_H_(*i*) = *V*_H_(3,4) = *V*_Pt_ is defined as

$$V_{\text{Pt}}=\left[1+\Delta_{H}\right]R_{H}\left(i\right)I_{\text{Ot}},$$ (1)

where *∆*_H_ is the correction factor to *R*_H_(*i*) to be determined in this analysis.

Next we describe the equivalent circuit model of the QHE device located in the central dashed-line region of Fig. 1. This model is based on that of Ricketts and Kemeny [18]. The device has contact pads that provide electrical access to the 2DEG at the source S*′*, the drain D*′*, and the potential pads 1*′* through 6*′*. Each contact pad is located at the end of an arm of the QHE device. Every arm in the equivalent circuit has an intrinsic resistor whose value is *R*_H_(*i*)/2. We assume that the device is homogeneous, i.e., that the quantized Hall resistances *R*_H_(*i*) are all measured on plateau regions, that their values are the same on all the Hall potential probe sets, and that they are all measured at the same magnetic flux density value. *R*_H_(*i*) can, however, vary with temperature [13] and current [14].

While *V*_Pt_ has been observed to vary with frequency [4–9], it is not clear whether this is due to a frequency dependence of *R*_H_(*i*), of *∆*_H_, or of both *R*_H_(*i*) and *∆*_H_. Calculations of the intrinsic impedance of the 2DEG due to the internal Hall capacitance across the QHE device [19], however, predict a negligible frequency dependence of *R*_H_(*i*) itself, implying a frequency dependence of *∆*_H_ arising from parasitic impedances in the ac QHRS. We therefore simplify the model, and assume that the dc values are appropriate for the *R*_H_(*i*)/2 resistances in the figure.

The symbols *r*_a_, *r*_b_, *r*_c_, and *r*_d_ in Fig. 1 represent real (in-phase) longitudinal resistances within the QHE device. Their measured dc values can vary with temperature [13] and current [14]. Sample probes normally used in dc QHE measurements have ten leads, with a pair of leads to the source contact pad S*′* and another pair to the drain contact pad D*′*. Only one lead of each pair carries the current, so the dc values of all four longitudinal resistances *r*_a_, *r*_b_, *r*_c_, and *r*_d_ can be obtained using four-terminal measurements.

In order to reduce the heat load on the liquid helium, sample probes for the ac QHE usually have a single coaxial lead to each of the eight contact pads. Therefore only *r*_b_ and *r*_c_ can be determined directly via four-terminal-pair ac measurements. For example, a four-terminal-pair ac longitudinal resistance measurement of *r*_b_ could be made by moving the Potential coaxial port from access position 3 to position 2 in Fig. 1, and measuring the ac longitudinal voltage *V_x_*(2,4).

$$V_{x}\left(2,4\right)=\left[1+\Delta_{24}\right]r_{b}\;I_{\text{Ot}},$$ (2)

where *∆*_24_ is the correction factor to *r*_b_ to be determined in this analysis. Values for *r*_a_ and *r*_d_ could be estimated from their dc *r*_a_/*r*_b_ and *r*_d_/*r*_c_ ratios if the measured *r*_b_/*r*_c_ ratio happens to be the same for both ac and dc measurements using the same sample probe during the same cool-down.

With one exception [20], the reported ac longitudinal resistances obtained from the real, in-phase components of the ac longitudinal voltage measurements are significantly larger than the dc longitudinal resistances in the same device under the same temperature and magnetic field conditions. The ac longitudinal resistances increase with increasing frequency of the applied current, and are of order 1 mΩ at 1592 Hz [4,5,21]. The large ac longitudinal voltages might be due to intrinsic frequency dependences of *r*_a_, *r*_b_, *r*_c_, and *r*_d_ within the device, to *∆*_24_, *∆*_46_, etc. corrections caused by parasitic impedances of the QHRS, or to both of them. Calculations of the kinetic inductance of the 2DEG and the magnetic inductance of the device [20] provide no plausible explanations via intrinsic impedance for significant frequency dependences of *r*_a_, *r*_b_, *r*_c_, and *r*_d_, suggesting that the frequency dependence of the ac longitudinal resistance is due to parasitic impedances of the QHRS, and therefore to the correction factors *∆*_24_, *∆*_46_, etc. However, we will assume the worst-case scenario in our numerical calculations, that is *r*_a_, *r*_b_, *r_c_*, and *r*_d_ are themselves frequency dependent and have 1 mΩvalues at 1592 Hz.

At some moment in time, a positive current *I*_a_ enters the 2DEG via device drain contact pad D*′* in Fig. 1, and current *I*_d_ exits the 2DEG via source contact pad S*′*. The magnetic flux density *B* is directed into the figure from above. Under these current and magnetic field conditions, the drain contact pad D*′* and the potential probe contact pads 1*′*, 3*′*, and 5*′* at the device periphery are at higher potentials than contact pads S*′*, 2*′*, 4*′*, and 6*′*. These current and flux density directions are chosen to be consistent with those we have used in earlier calculations [19,22–23].

Potentials at the contact pads S*′*, 1*′* through 6*′*, and D*′* are produced by arrays of voltage generators, where each voltage generator *V*_AB_ is located between a pair of arms A and B of the equivalent circuit. The voltages are defined as

$$V_{\text{AB}}\equiv \frac{R_{H}\left(i\right)}{2}\left|I_{A}\pm I_{B}\right|,$$ (3)

where *I*_A_ and *I*_B_ are the magnitudes of the current flowing in arms A and B. The currents *I*_A_ and *I*_B_ within the absolute quantity sign of Eq. (3) are added if they both enter or both leave the voltage generator, and are subtracted if one current enters and the other current leaves the generator. For example $V_{1D}=\;[R_{H}(i)/2]\left|I_{a}-I_{C_{1}}\right|$. The voltages generated are functions of *R*_H_(*i*); therefore their values can vary with temperature [13] and current [14] (and also possibly with frequency).

Diamond-shaped voltage generator arrays of Ricketts and Kemeny [18] are employed in the equivalent circuit of the QHE device, rather than the ring-shaped voltage generator arrays introduced later by Delahaye [24] and then subsequently used by Jeffery, Elmquist, and Cage [25]. Although both arrays give essentially identical results [22], the calculations are much simpler with the diamond arrays when longitudinal resistances are included in the circuits [22]. We therefore use diamond arrays.

For clarity, the voltage generators are indicated in the figure as batteries, with positive terminals oriented to give the correct potentials along each arm at the instant considered. The ac currents alternate direction, so the voltage generators reverse sign each half cycle. Thus, for the part of the period in which the currents flow in the directions indicated in Fig. 1, the voltage generators have the polarities shown. Half a period later the currents change direction, and all the voltage generators reverse polarities.

The QHE device is mounted on a sample holder at the bottom of the sample probe. The QHE device and the sample holder are located within the shaded region of Fig. 1. Thin wires connect the device contact pads S*′*, 1*′* through 6*′*, and D*′* to coaxial leads which extend to room temperature access points S, 1 through 6, and D located outside the sample probe (but still within the ac QHRS). Each arm of the equivalent circuit has a resistance *r*_S_, *r*_1_ through *r*_6_, or *r*_D_. This resistance includes the contact resistance to the 2DEG, the wire resistance connecting a contact pad on the device to a coaxial lead, and the inner conductor resistance of that coaxial lead. The inner conductor lead resistances vary with the liquid helium level in the sample probe. They can be measured pair-wise (using access points S, 1 through 6, and D) as a function of liquid helium level via two-terminal dc resistance measurements by temporarily replacing the QHE device with electrical shorts at positions S*′*, 1*′* through 6*′*, and D*′*. The cooled inner conductor coaxial lead resistances are typically each about 1 Ω in ac quantized Hall resistance standards. The outer conductor coaxial lead resistances depend on the type of coaxial cable, and their values also vary with liquid helium level. Typical values range between about 0.1 Ωand 1 Ω in ac quantized Hall resistance experiments.

Each sample probe lead has an inductance *L*_S_, *L*_1_ through *L*_6_, or *L*_D_, that is electrically connected in series with the lead resistances *r*_S_, *r*_1_ through *r*_6_, or *r*_D_, producing lead impedances *z*_S_, *z*_1_ through *z*_6_, or *z*_D_, where *z*_S_ = *r*_S_ + j*ωL*_S_. Due to severe space limitations in the figure, these impedances are unconventionally drawn as resistors within rectangles. The inductance of each coaxial lead of a typical ac QHE sample probe is about 1 × 10^−6^ H. We assume that the bonding pad wires are thick enough to not vibrate in the magnetic field when applied ac currents flow through them [4], but the out-of-phase “inductance” generated by this vibration [4] could be included in the lead inductances if necessary.

The eight coaxial leads, labeled S, 1 through 6, and D, each have an inner and an outer conductor. The outer conductors of the coaxial leads are connected together outside the sample probe to help satisfy the four-terminal-pair measurement conditions. As mentioned earlier, the outer conductors of these leads act as electrical shields, and are represented schematically as thick lines in Fig. 1. (Other outer conductors of the ac QHRS also contribute to the thick lines.)

Large capacitances-to-shield, labeled as *C*_S_, *C*_1_ through *C*_6_, and *C*_D_, exist between the inner and outer conductors of these coaxial leads. The open-circuit capacitances can be individually measured at access points S, 1 through 6, and at D as a function of liquid helium level by temporarily removing the QHE device from the sample probe at the points S*′*, 1*′* through 6*′*, and D*′*. The capacitance-to-shield of each coaxial lead in typical ac QHE sample probes is about 250 pF, but it should be reduced to about 100 pF (1 × 10^−10^ F) in a short sample probe being designed at NIST.

A predominately 90° out-of-phase current $I_{C_{S}}$, $I_{C_{1}}$ through $I_{C_{6}}$, or $I_{C_{D}}$ flows through each coaxial lead. These currents, and all the other currents in Fig. 1, have the correct signs for their dominant phase components in the half-cycle under consideration. This is verified in Sec. 5, where it is found that all currents shown in the figure have positive signs for their major components.

The coaxial leads are not the only sources of capacitances-to-shield. There are also additional contributions from the QHE device—sample holder combination and the electrical shielding surrounding them. These additional capacitances-to-shield are labeled *C*_A_ and *C*_B_ in Fig. 1, where they are placed at either end of the QHE device. (Note that rather than explicitly using *C*_A_ and *C*_B_, one-eighth of the additional capacitances *C*_A_ + *C*_B_ could instead be added to each of the eight coaxial lead capacitances *C*_S_, *C*_1_ through *C*_6_, and *C*_D_, but that would make the coaxial lead capacitance notation very confusing.)

The additional capacitances *C*_A_ and *C*_B_ can be determined by two methods. In the first method the magnetic field is adjusted so the QHE device is on a QHE plateau. The external coaxial leads from the bridge are removed from the Drive and Inner/Outer ports of the ac QHRS, and an applied voltage signal is placed across the inner and outer conductors of the Drive port. A measured voltage signal appears across the inner and outer conductors of coaxial leads S, D, 1, 3, and 5 for the magnetic field direction assumed in Fig. 1, so these particular coaxial leads draw most of the 90° out-of-phase current. Therefore the measured total capacitance-to-shield *C*_T_ is approximately *C*_T_(*B*) ≈ *C*_1_ + *C*_3_ + *C*_5_ + *C*_D_ + *C*_A_, and the value of *C*_A_ can be obtained by subtracting the value of *C*_1_ + *C*_3_ + *C*_5_ + *C*_D_ from *C*_T_(*B*). The magnetic field is reversed. Then *C*_T_(−*B*) ≈ *C*_2_ + *C*_4_ + *C*_6_ + *C*_S_ + *C*_B_ when the voltage signal is placed across the inner and outer conductors at the Inner/Outer port, thus yielding the value of *C*_B_. In the second method the magnetic flux density *B* is reduced to zero. The quantum Hall voltages disappear, so the voltage generators can be replaced in the circuit by electrical shorts. The QHE device now behaves like a two-dimensional sheet resistance, and the *R*_H_(*i*)/2 resistances located at the source and drain ends of the QHE device in Fig 1 are zero. Longitudinal resistances *r*_a_, *r*_b_, *r*_c_, and *r*_d_ become much larger than they were when on a QHE plateau. Their values can be obtained by four-terminal resistance measurements in a dc sample probe. The *R*_H_(*i*)/2 resistances of the six QHE side arms are replaced by much smaller resistances whose values can be obtained from two-terminal measurements via room temperature access points S, 1 through 6, and D once the appropriate lead and longitudinal resistances are subtracted. An applied voltage signal placed across the inner and outer conductors of the Drive port would cause a voltage signal to appear across the inner and outer conductors of all capacitances-to-shield. Thus the total capacitance-to-shield is given by the expression *C*_T_ = *C*_S_ + *C*_1_ + *C*_2_ + *C*_3_ + *C*_4_ + *C*_5_ + *C*_6_ + *C*_D_ + *C*_A_ + *C*_B_, where *C*_A_ ≈ *C*_B_ if the QHE device, the sample holder, and the bonding wires between them are all symmetrically arranged. We expect both *C*_A_ and *C*_B_ will be about 1 pF or smaller in the NIST sample probes.

The equivalent circuit accounts for leakage currents between the ac QHRS′s inner conductors and the shields via resistances $r_{K_{A}}$ and $r_{K_{B}}$ located on either side of the QHE device. Rather large voltages are used when measuring leakage resistances, so it would be safest to temporarily replace the device with shorts when measuring the total open-circuit leakage resistance *r*_Lk_ at access point S, 1 through 6, or D. If the leakage resistances are symmetrically distributed, then $r_{K_{A}}$ ≈ $r_{K_{B}}$ ≈ 2*r*_LK_. (Their values are large compared with the lead resistances, so they are essentially connected in parallel within the circuit.) The NIST sample probes will be constructed so these leakage resistances are very large; $r_{K_{A}}$ and $r_{K_{B}}$ should be at least 10^14^ Ω, but in the numerical examples of this paper we will assume 10^12^ Ω arising from dirty coaxial connectors.

The capacitances, inductances, and leakage resistances of Fig. 1 contribute parasitic impedances to measurements of the ac QHRS. These capacitances, inductances, and leakage resistances are drawn as discrete circuit elements. In reality they are distributed within the standard. They could, in principle, be better represented. For example, we could replace capacitance-to-shield *C*_1_ with a capacitor of value *C*_1_/2, and place a second capacitor of value *C*_1_/2 and a series-connected outer shield impedance $z_{1}^{\prime }$ between the other side of circuit element *z*_1_ at point 1*′* and the first *C*_1_/2 capacitor. This distributed impedance would, however, greatly complicate the circuit analyses, with little gain in accuracy. (Our discrete elements circuit over-emphasizes the capacitance-to-shield currents if *z*_1_ ≥ $z_{1}^{\prime }$ and gives the same capacitance-to-shield currents if *z*_1_ = $z_{1}^{\prime }$.)

This completes the description of the equivalent circuit. The next section analyzes the circuit.

## 5. Analysis of the Single-Series “Normal” Circuit

Kirchoff′s rules are used to sum the currents at branch points and the voltages around loops to obtain exact algebraic equations for the equivalent electrical circuit shown in Fig. 1. We refer to this circuit as single-series “normal”: single-series because there is just one current lead connected to the source contact pad S*′*, and another current lead connected to the drain pad D*′* of the QHE device; and “normal” because the Hall voltage leads are connected to the central arms 3 and 4 of the device.

### 5.1 Exact Single-Series “Normal” Equations

Finding the exact algebraic equations for all the currents, and for the correction factor *∆*_H_ to the quantum Hall voltage, as defined by Eq. (1), is rather difficult because there are many coupled equations, especially for the multi-series circuits [24] examined later in this paper. All the solutions of this paper were independently derived by each author, and shown to be identical. Then each author independently used computer software to obtain identical numerical results for several test cases. It is important to obtain the exact solutions, rather than initially guess approximate solutions, because the frequency dependent effects we are trying to minimize or eliminate are small, but significant. The results are presented here in order to spare others the task of deriving them.

To simplify the final algebraic expressions, we define some substitutions of variables, and substitutions of substitutions. The particular substitutions depend on the choice of loops. For example, the variables *A* and *B* listed below result from a voltage loop around the path *C*_S_, S*′*, *C*_B_, and back through the shield to *C*_S_. This gives the equation $-\frac{1}{j\omega C_{S}}I_{C_{S}}+z_{S}I_{S}+\frac{1}{j\omega C_{B}}I_{C_{B}}=0,\;\text{so}\;I_{C_{B}}=\frac{C_{B}}{C_{S}}I_{C_{S}}-j\omega C_{B}z_{S}I_{S},\;\text{or}\;I_{C_{B}}=AI_{C_{S}}-BI_{S}$. Let

$$A=\frac{C_{B}}{C_{S}}$$ (4a)

$$B=j\omega C_{B}z_{S}$$ (4b)

$$C=\frac{1}{j\omega C_{B}r_{K_{B}}}$$ (4c)

$$D=\frac{1}{\left[1+j\omega C_{6}\left(R_{H}+z_{6}\right)\right]}\frac{C_{6}}{C_{B}}$$ (4d)

$$E=\frac{j\omega C_{6}r_{d}}{\left[1+j\omega C_{6}\left(R_{H}+z_{6}\right)\right]}$$ (4e)

$$F=\frac{1}{\left[1+j\omega C_{5}z_{5}\right]}\frac{C_{5}}{C_{B}}$$ (4f)

$$G=\frac{j\omega C_{5}\left(R_{H}+r_{d}\right)}{\left[1+j\omega C_{5}z_{5}\right]}$$ (4g)

$$H=\frac{\left[1+j\omega C_{5}\left(R_{H}+z_{5}\right)\right]}{\left[j\omega C_{5}\left(R_{H}-r_{c}\right)\right]}$$ (4h)

I=1+C+D+F(1−H) (4i)

J=E+G(1−H) (4j)

K=I+J(1+C) (4k)

$$L=\frac{AK}{\left[1+J+K\left(A+B\right)\right]}$$ (4l)

$$M=\frac{j\omega C_{3}R_{H}}{\left[1+j\omega C_{3}z_{3}\right]}$$ (4m)

$$N=\frac{j\omega C_{2}r_{b}}{\left[1+j\omega C_{2}\left(R_{H}+z_{2}\right)\right]}$$ (4n)

$$O=\frac{j\omega C_{1}\left(R_{H}+r_{b}\right)}{\left[1+j\omega C_{1}z_{1}\right]}$$ (4o)

$$P=\frac{\left(R_{H}+r_{a}\right)}{r_{K_{A}}}$$ (4p)

$$Q=\frac{\left[1+j\omega C_{2}z_{2}\right]}{\left[j\omega C_{2}r_{K_{A}}\right]}$$ (4q)

$$R=j\omega C_{A}r_{K_{A}}$$ (4r)

$$S=\frac{C_{D}}{C_{A}}$$ (4s)

$$T=j\omega C_{D}z_{D}.$$ (4t)

We express all currents, and the quantum Hall voltage, in terms of *I*_Ot_ because that is the current that enters the ac reference standard (not shown in Fig. 1). Three of the current solutions are trivial because of the four-terminal-pair definition [15,16] listed in Sec. 4

$$I_{\text{Dr}}=I_{\text{Pt}}=I_{C_{4}}=0.$$ (5a)

The remaining exact equations for the single-series “normal” circuit currents are

$$I_{S}=LI_{\text{Ot}}$$ (5b)

$$I_{C_{S}}=I_{\text{Ot}}-I_{S}$$ (5c)

$$I_{C_{B}}=AI_{C_{S}}-BI_{S}$$ (5d)

$$I_{K_{B}}=CI_{C_{B}}$$ (5e)

$$I_{d}=I_{S}-I_{C_{B}}-I_{K_{B}}$$ (5f)

$$I_{C_{6}}=DI_{C_{B}}-EI_{d}$$ (5g)

$$I_{C_{5}}=-FI_{C_{B}}+GI_{d}$$ (5h)

$$I_{c}=HI_{C_{5}}$$ (5i)

$$I_{C_{3}}=MI_{c}$$ (5j)

$$I_{b}=I_{c}+I_{C_{3}}$$ (5k)

$$I_{C_{2}}=NI_{b}$$ (5l)

$$I_{C_{1}}=OI_{b}$$ (5m)

$$I_{a}=I_{b}+I_{C_{1}}+I_{C_{2}}$$ (5n)

$$I_{K_{A}}=PI_{a}+QI_{C_{2}}$$ (5o)

$$I_{C_{A}}=RI_{K_{A}}$$ (5p)

$$I_{D}=I_{a}+I_{K_{A}}+I_{C_{A}}$$ (5q)

$$I_{C_{D}}=SI_{C_{A}}+TI_{D}$$ (5r)

$$I_{D_{r}}=I_{D}+I_{C_{D}}.$$ (5s)

The exact equation for the quantum Hall voltage is obtained by summing the voltages between the inner conductors of the Detector coaxial port and the Potential coaxial port. Taking the path through arm 4, voltage generators *V*_c4_ and *V*_c3_, and arm 3 we find that

$$V_{H}\left(3,4\right)=R_{H}I_{c}-z_{3}I_{C_{3}},$$ (6a)

which can also be expressed in the form

$$V_{H}\left(3,4\right)=\left[1+\Delta_{34}\right]R_{H}I_{\text{Ot}}$$ (6b)

by using Eqs. (4) and (5). An approximate solution in this form will be given in Eq. (11).

### 5.2 A Numerical Example

Contributions of the parasitic impedance within the ac QHRS to the measured value of *V*_H_(3,4) can be investigated by using numerical examples in Eqs. (5) and (6). We assign cardinal numbers to circuit element values to emphasize that the results are not intended to provide corrections to existing experimental data because the effects of wire-to-wire capacitances are not included at this intermediate stage of the analysis.

Both the *i* = 2 (12 906.4 Ω) and *i* = 4 (6 453.2 Ω) plateaus have been measured in ac experiments, so let *R*_H_ = 10 000 Ω. The cardinal values we use are

$$R_{H}=10^{4}\;\Omega$$ (7a)

$$r_{S}=r_{1}=r_{2}=r_{3}=r_{4}=r_{5}=r_{6}=r_{D}=1\;\Omega$$ (7b)

$$r_{a}=r_{b}=r_{c}=r_{d}=10^{-3}\;\Omega$$ (7c)

$$r_{K_{A}}=r_{K_{B}}=10^{12}\;\Omega$$ (7d)

$$C_{S}=\;C_{1}=C_{2}=C_{3}=C_{4}=C_{5}=C_{6}=C_{D}=10^{-10}F$$ (7e)

$$C_{A}=C_{B}=10^{-12}F$$ (7f)

$$L_{S}=L_{1}=L_{2}=L_{3}=L_{4}=L_{5}=L_{6}=L_{D}=10^{-6}H$$ (7g)

$$\omega =10^{4}\;\text{rad/s}.$$ (7h)

Note that the 100 pF capacitances-to-shield values of Eq. (7e) may be close to those that will be obtained in the short NIST sample probe, but typical ac probes have values around 250 pF.

The numerical results for the currents of Eqs. (5) are

$$I_{C_{S}}=\{-[1.0\times 10^{-8}]+j[1.0\times 10^{-6}]\}I_{\text{Ot}}$$ (8a)

$$I_{S}=\left\{\left[1.00000\right]-j\left[1.0\times 10^{-6}\right]\right\}I_{\text{Ot}}$$ (8b)

$$I_{C_{B}}=\left\{-\left[2.0\times 10^{-13}\right]+j\left[2.0\times 10^{-11}\right]\right\}I_{\text{Ot}}$$ (8c)

$$I_{K_{B}}=\left\{\left[2.0\times 10^{-15}\right]+j\left[2.0\times 10^{-17}\right]\right\}I_{\text{Ot}}$$ (8d)

$$I_{d}=\{[1.00000]-j[1.0\times 10^{-6}]\}I_{\text{Ot}}$$ (8e)

$$I_{C_{6}}=\{-[1.0\times 10^{-11}]+j[1{.0\times 10}^{-9}]\}I_{\text{Ot}}$$ (8f)

$$I_{C_{5}}=\{[2.0\times 10^{-8}]+j[0.01000]\}I_{\text{Ot}}$$ (8g)

$$I_{c}=\{[1.00000]+j[0.01000]\}I_{\text{Ot}}$$ (8h)

$$I_{C_{3}}=\{-[1.0\times 10^{-4}]+j[0.01000]\}I_{\text{Ot}}$$ (8i)

$$I_{b}=\{[0.99990]+j[0.02000]\}I_{\text{Ot}}$$ (8j)

$$I_{C_{2}}=\{-[1.0\times 10^{-11}]+j[1{.0\times 10}^{-9}]\}I_{\text{Ot}}$$ (8k)

$$I_{C_{1}}=\{-[2.0\times 10^{-4}]+j[0.01000]\}I_{\text{Ot}}$$ (8l)

$$I_{a}=\{[0.99970]+j[0.03000]\}I_{\text{Ot}}$$ (8m)

$$I_{K_{A}}=\{[1.0\times 10^{-8}]+j[3{.0\times 10}^{-10}]\}I_{\text{Ot}}$$ (8n)

$$I_{C_{A}}=\{-[3.0\times 10^{-6}]+j[1{.0\times 10}^{-4}]\}I_{\text{Ot}}$$ (8o)

$$I_{D}=\{[0.99970]+j[0.03010]\}I_{\text{Ot}}$$ (8p)

$$I_{C_{D}}=\{-[3.0\times 10^{-4}]+j[0.01000]\}I_{\text{Ot}}$$ (8q)

$$I_{\text{Dr}}=\{[0.99940]+j[0.04010]\}I_{\text{Ot}}.$$ (8r)

The 90°out-of-phase (j) parts of shunt currents $I_{C_{5}},I_{C_{3}},I_{C_{1}},I_{C_{D}},\;$ and $I_{C_{A}}$, are much larger than for shunt currents $I_{C_{2}},I_{C_{6}},I_{C_{S}},\;$ and $I_{C_{B}}$ because contact pads 5*′*, 3*′*, 1*′*, and D*′* are all near the quantum Hall potential, rather than near the shield potential. A 1 % out-of-phase current passes through each of the coaxial cable capacitances *C*_5_, *C*_3_, *C*_1_, and *C*_D_ in this example. That is not necessarily a problem if the bridge Drive can provide this extra 4 % of out-of-phase current to *I*_Dr_.

Expressing Eq. (6a) in the form of Eq. (6b), we find that

$$V_{H}(3,4)=\{1+[5.0\times 10^{-8}]+j[0.01000]\}R_{H}I_{\text{Ot}},$$ (9a)

$$\Delta_{34}=\{[5.0\times 10^{-8}]+j[0.01000]\}.$$ (9b)

The 5 × 10^−8^ in-phase correction to *R*_H_ is too large compared with the desired 1 × 10^−8^ *R*_H_ absolute accuracy, but even worse, there is a 1 % contribution to *V*_H_(3,4) in the 90° out-of-phase j term. Auxiliary balances in the NIST high precision ac bridges are not capable of providing out-of-phase adjustment signals larger than 5 × 10^−4^ *R*_H_, so the 1 % out-of-phase signal is unacceptable. We next list the approximate solutions to show the source of this problem.

### 5.3 Approximate Single-Series “Normal” Solutions

Many of the terms in the following approximate solutions were obtained by algebraically finding the dominant contributions to the exact equations. Other terms were found by “educated guesses” and “trial-and-error”. We verified all the terms by changing the values of relevant circuit element components in the computer programs. The following approximate solutions give numerical results that agree with the results from the exact solutions to within at least two significant figures for both the real and imaginary parts of the numerical results. Other terms may need to be added to these approximate equations if the circuit components have values significantly different from those listed in Eqs. (7).

$$I_{C_{S}}\approx \{-[\omega^{2}C_{S}(C_{5}+C_{6})R_{H}r_{c}-\omega^{2}C_{S}C_{S}r_{S}r_{S}+\omega^{2}C_{S}L_{S}]+j[\omega C_{S}r_{S}]\}I_{\text{Ot}}$$ (10a)

$$I_{S}\approx \{1-j[\omega C_{S}r_{S}]\}I_{\text{Ot}}$$ (10b)

$$I_{C_{B}}\approx \{-[\omega^{2}C_{B}(C_{5}+C_{6})R_{H}r_{c}]+j[\omega C_{B}(r_{c}+r_{d})]\}I_{\text{Ot}}$$ (10c)

$$I_{K_{B}}\approx \left\{\left[\frac{\left(r_{c}+r_{d}\right)}{r_{K_{B}}}\right]+j\left[\omega (C_{5}+C_{6})\frac{R_{H}r_{c}}{r_{K_{B}}}\right]\right\}I_{\text{Ot}}$$ (10d)

$$I_{d}\approx \{1-j[\omega C_{S}r_{S}]\}I_{\text{Ot}}$$ (10e)

$$I_{C_{6}}\approx \{-[\omega^{2}C_{5}C_{6}R_{H}r_{c}]+j[\omega C_{6}r_{c}]\}I_{\text{Ot}}$$ (10f)

$$I_{C_{5}}\approx \{[\omega^{2}C_{S}C_{5}R_{H}r_{S}+\omega^{2}C_{5}C_{5}R_{H}r_{5}]+j\left[\omega C_{5}R_{H}\right]\}I_{\text{Ot}}$$ (10g)

$$I_{c}\approx I_{\text{Ot}}+I_{C_{5}}-I_{C_{S}}$$ (10h)

$$I_{C_{3}}\approx \{-[\omega^{2}C_{3}C_{5}R_{H}R_{H}]+j\left[\omega C_{3}R_{H}\right]\}I_{\text{Ot}}$$ (10i)

$$I_{b}\approx I_{\text{Ot}}+I_{C_{5}}+I_{C_{3}}$$ (10j)

$$I_{C_{2}}\approx \{-[\omega^{2}C_{2}(C_{3}+C_{5}-C_{2})R_{H}r_{b}]+j[\omega C_{2}r_{b}]\}I_{\text{Ot}}$$ (10k)

$$I_{C_{1}}\approx \{-[\omega^{2}C_{1}(C_{3}+C_{5})R_{H}R_{H}]+j[\omega C_{1}R_{H}]\}I_{\text{Ot}}$$ (10l)

$$I_{a}\approx I_{\text{Ot}}+I_{C_{5}}+I_{C_{3}}+I_{C_{1}}$$ (10m)

$$I_{K_{A}}\approx \left\{\left[\frac{R_{H}}{r_{K_{A}}}\right]+j\left[\omega \left(C_{1}+C_{3}+C_{5}\right)\frac{R_{H}R_{H}}{r_{K_{A}}}\right]\right\}I_{\text{Ot}}$$ (10n)

$$I_{C_{A}}\approx \{-[\omega^{2}C_{A}(C_{1}+C_{3}+C_{5})R_{H}R_{H}]+j\left[\omega C_{A}R_{H}\right]\}I_{\text{Ot}}$$ (10o)

$$I_{D}\approx I_{\text{Ot}}+I_{C_{5}}+I_{C_{3}}+I_{C_{1}}+I_{C_{A}}$$ (10p)

$$I_{C_{D}}\approx \{-[\omega^{2}C_{D}(C_{1}+C_{3}+C_{5})R_{H}R_{H}]+j\left[\omega C_{D}R_{H}\right]\}I_{\text{Ot}}$$ (10q)

$$I_{\text{Dr}}\approx I_{\text{Ot}}+I_{C_{5}}+I_{C_{3}}+I_{C_{1}}+I_{C_{A}}+I_{C_{D}}.$$ (10r)

Expressing Eq. (6a) in the form (6b),

$$\begin{array}{l} \Delta_{Η}\approx \{[\omega^{2}C_{S}C_{5}R_{H}r_{S}+\omega^{2}C_{3}C_{5}R_{H}r_{3}+\omega^{2}C_{5}C_{5}R_{H}r_{5}\\ -\omega^{2}C_{S}C_{S}r_{S}r_{S}]+[\omega^{2}C_{S}\left(C_{5}+C_{6}\right)R_{H}r_{c}+\omega^{2}C_{S}L_{S}\\ +\omega^{2}C_{3}L_{3}]+j[\omega C_{5}R_{H}-\omega C_{S}r_{S}-\omega C_{3}r_{3}]\}. \end{array}$$ (11)

We see from Eq. (11) that sample probe lead 5 is the dominant source of the 1 % out-of-phase component of the quantum Hall voltage signal. The next subsection investigates the effect of removing this lead.

### 5.4 Disconnecting Sample Probe Lead 5

Equation (11) predicts that the out-of-phase term j[*ω C*_5_*R*_H_] in the expression for *∆*_34_ can be reduced by disconnecting coaxial cable 5 at position 5*′*, where 5*′* is either located at the potential contact pad on the QHE device, or at an intermediate contact point in the sample holder. There is a capacitance *C*_5_*_′_* between the QHE device and the shield that replaces capacitance *C*_5_ in Fig. 1. Also, a shield impedance *z*_5_*_′_* replaces the lead impedance *z*_5_.

The most significant terms of Eq. (11) are now

$$\begin{array}{l} \Delta_{34}\approx \{[\omega^{2}C_{S}L_{S}+\omega^{2}C_{3}L_{3}]+\\ j[\omega C_{5\prime }\;R_{H}-\omega C_{S}r_{S}-\omega C_{3}r_{3}]\}. \end{array}$$ (12)

If we assume in the numerical examples that

$$C_{5\prime }=C_{A}=C_{B}=1\;\text{pF}$$ (13)

$$r_{5\prime }=r_{5}=1\;\Omega .$$ (14)

Then

$$\Delta_{34}=\{[2.0\times 10^{-8}]+j[9{.8\times 10}^{-5}]\}$$ (15)

when the coaxial lead capacitances are all 100 pF, and

$$\Delta_{34}=\{[5.0\times 10^{-8}]+j[9{.5\times 10}^{-5}]\}$$ (16)

when they are 250 pF. All experiments which have measured ac values of *V*_H_(3,4) have had to remove coaxial lead 5 because of the effects due to the large capacitance-to-shield *C*_5_ presented above.

Equations (11) or (12) might be used to apply corrections to the experimental data in order to reduce the 5 × 10^−8^ in-phase error in *R*_H_*I*_Ot_. However, there are several points of concern: (a) our approximate and exact equations do not include the effects of wire-to-wire capacitances, and these may be significant; (b) the out-of-phase component of *V*_H_(3,4) has been reduced to about 1 × 10^−4^ *R*_H_*I*_Ot_ by removing lead 5, but great care must be taken to correct for the in-phase (phase defect) contributions of the bridge components used to null the out-of-phase signal because these in-phase (phase defect) signals can be unintentionally added to the real, second-order terms of the in-phase component of *V*_H_(3,4) in Eqs. (11) or (12) that vary with *ω*^2^; and (c) it is not trivial to measure the value of *C*_5_*_′_* in order to apply the correction with lead 5 disconnected.

We will not consider further the single-series “normal” circuit as a viable ac QHRS candidate because lead 5 must be disconnected, and that violates one of our desired goals.

## 6. Analysis of the Single-Series “Offset” Circuit

**Fig. 2:**
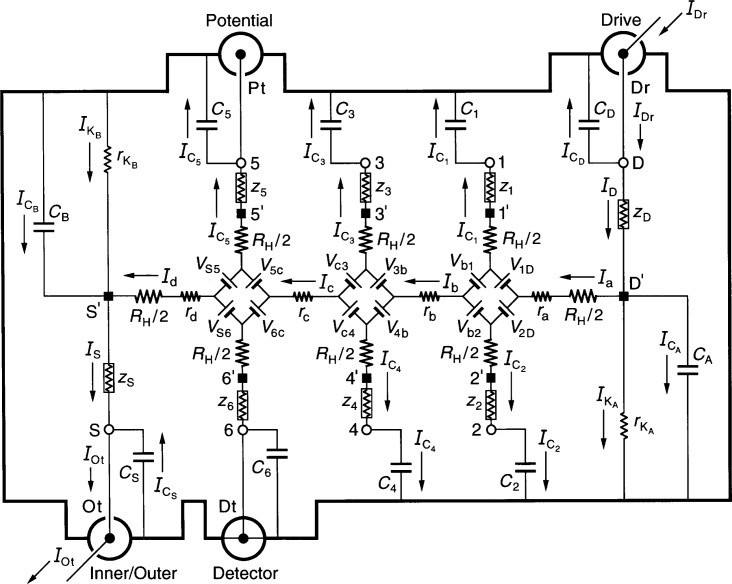
An equivalent electrical circuit representation of an ac QHE resistance standard with single-series “offset” connections to the device. See Sec. 6 for the circuit analysis.

Figure 2 shows an equivalent electrical circuit representation of an ac QHRS using single-series “offset” connections to the QHE device. It is single-series because there is just one current lead connected to the source contact pad S*′*, and another current lead connected to the drain pad D*′* of the QHE device, and “offset” because the Hall voltage leads are connected to the off-center arms 5 and 6 of the device. Arms 5 and 6 are closest to the low potential end of the device at S*′*, and nearest to the ac reference resistor (not shown in the figure). Those arms were chosen in an attempt to reduce the effects of shunt currents through *I*_C5_ that we found in Sec. 5.

### 6.1 Exact Single-Series “Offset” Equations

To simplify the final algebraic expressions, we again define some intermediate substitutions of variables, and substitutions of substitutions. Let

$$A=\frac{C_{B}}{C_{S}}$$ (17a)

$$B=j\omega C_{B}z_{S}$$ (17b)

$$C=\frac{1}{j\omega C_{B}r_{K_{B}}}$$ (17c)

$$D=j\omega C_{S}z_{S}$$ (17d)

$$E=j\omega C_{S}r_{d}$$ (17e)

F=BE(1+C) (17f)

G=AE(1+C) (17g)

H=1+D+E+F+G (17h)

$$I=\frac{j\omega C_{5}R_{H}}{\left[1+j\omega C_{5}z_{5}\right]}$$ (17i)

$$J=\frac{j\omega C_{4}r_{c}}{\left[1+j\omega C_{4}\left(R_{H}+z_{4}\right)\right]}$$ (17j)

$$K=\frac{j\omega C_{3}\left(R_{H}+r_{c}\right)}{\left[1+j\omega C_{3}z_{3}\right]}$$ (17k)

$$L=\frac{j\omega C_{2}r_{b}}{\left[1+j\omega C_{2}\left(R_{H}+z_{2}\right)\right]}$$ (17l)

$$M=\frac{\left[\frac{C_{2}}{C_{4}}+j\omega C_{2}z_{4}\right]}{\left[1+j\omega C_{2}(R_{H}+z_{2})\right]}$$ (17m)

$$N=\frac{j\omega C_{1}r_{b}}{\left[1+j\omega C_{1}z_{1}\right]}$$ (17n)

$$O=\frac{\left[\frac{C_{1}}{C_{3}}+j\omega C_{1}(R_{H}+z_{3})\right]}{\left[1+j\omega C_{1}z_{1}\right]}$$ (17o)

$$P=\frac{\left(R_{H}+r_{a}\right)}{r_{K_{A}}}$$ (17p)

$$Q=\frac{\left[1+j\omega C_{2}z_{2}\right]}{\left[j\omega C_{2}r_{K_{A}}\right]}$$ (17q)

$$R=j\omega C_{A}r_{K_{A}}$$ (17r)

$$S=\frac{C_{D}}{C_{A}}$$ (17s)

$$T=j\omega C_{D}z_{D}.$$ (17t)

Three of the current solutions are trivial because of the four-terminal-pair definition [15,16]

$$I_{\text{Dt}}=I_{\text{Pt}}=I_{C_{6}}=0.$$ (18a)

The remaining exact equations for the single-series “offset” circuit currents are

$$I_{S}=\frac{\left(1+G\right)}{H}I_{\text{Ot}}$$ (18b)

$$I_{C_{S}}=I_{\text{Ot}}-I_{S}$$ (18c)

$$I_{C_{B}}=AI_{C_{S}}-BI_{S}$$ (18d)

$$I_{K_{B}}=CI_{C_{B}}$$ (18e)

$$I_{d}=I_{S}-I_{C_{B}}-I_{K_{B}}$$ (18f)

$$I_{C_{5}}=II_{d}$$ (18g)

$$I_{c}=I_{d}+I_{C_{5}}$$ (18h)

$$I_{C_{4}}=JI_{c}$$ (18i)

$$I_{C_{3}}=KI_{c}$$ (18j)

$$I_{b}=I_{c}+I_{C_{3}}+I_{C_{4}}$$ (18k)

$$I_{C_{2}}=LI_{b}+MI_{C_{4}}$$ (18l)

$$I_{C_{1}}=NI_{b}+OI_{C_{3}}$$ (18m)

$$I_{a}=I_{b}+I_{C_{1}}+I_{C_{2}}$$ (18n)

$$I_{K_{A}}=PI_{a}+QI_{C_{2}}$$ (18o)

$$I_{C_{A}}=RI_{K_{A}}$$ (18p)

$$I_{D}=I_{a}+I_{K_{A}}+I_{C_{A}}$$ (18q)

$$I_{C_{D}}=SI_{C_{A}}+TI_{D}$$ (18r)

$$I_{\text{Dr}}=I_{D}+I_{C_{D}}.$$ (18s)

The exact equation for the quantum Hall voltage is obtained by summing the voltages between the inner conductors of the Detector coaxial port and the Potential coaxial port. Taking the path through arm 6, voltage generators *V*_S6_ and *V*_S5_, and arm 5 we find that

$$V_{H}(5,6)=R_{H}I_{d}-z_{5}I_{C_{5}},$$ (19a)

which can also be expressed in the form

$$V_{H}(5,6)=\left[1+\Delta_{56}\right]R_{H}I_{\text{Ot}}.$$ (19b)

### 6.2 A Numerical Example

We investigate the parasitic impedance contributions of the ac QHRS on the measured value of *V*_H_(5,6) by using the cardinal numbers listed in Eqs. (7) in Eqs. (18) and (19). The numerical results for the currents are

$$I_{C_{s}}=\left\{-[1.0\times 10^{-8}\right]+j[1{.0\times 10}^{-6}]\}I_{\text{Ot}}$$ (20a)

$$I_{S}=\{[1.00000]-j[1{.0\times 10}^{-6}]\}I_{\text{Ot}}$$ (20b)

$$I_{C_{B}}=\{[1.0\times 10^{-17}]+j[1{.0\times 10}^{-11}]\}I_{\text{Ot}}$$ (20c)

$$I_{K_{B}}=\{[1.0\times 10^{-15}]-j[1{.0\times 10}^{-21}]\}I_{\text{Ot}}$$ (20d)

$$I_{d}=\{[1.00000]-j[1{.0\times 10}^{-6}]\}I_{\text{Ot}}$$ (20e)

$$I_{C_{5}}=\{[2.0\times 10^{-8}]+j[0.01000]\}I_{\text{Ot}}$$ (20f)

$$I_{c}=\{[1.00000]+j[0.01000]\}I_{\text{Ot}}$$ (20g)

$$I_{C_{4}}=\{[2.0\times 10^{-15}]+j[1{.0\times 10}^{-9}]\}I_{\text{Ot}}$$ (20h)

$$I_{C_{3}}=\{-[1.0\times 10^{-4}]+j[0.01000]\}I_{\text{Ot}}$$ (20i)

$$I_{b}=\{[0.99990]+j[0.02000]\}I_{\text{Ot}}$$ (20j)

$$I_{C_{2}}=\{[3.0\times 10^{-15}]+j[2{.0\times 10}^{-9}]\}I_{\text{Ot}}$$ (20k)

$$I_{C_{1}}=\{-[2.0\times 10^{-4}]+j[0.01000]\}I_{\text{Ot}}$$ (20l)

$$I_{a}=\{[0.99970]+j[0.03000]\}I_{\text{Ot}}$$ (20m)

$$I_{K_{A}}=\{[1.0\times 10^{-8}]+j[3{.0\times 10}^{-10}]\}I_{\text{Ot}}$$ (20n)

$$I_{C_{A}}=\{-[3.0\times 10^{-6}]+j[1{.0\times 10}^{-4}]\}I_{\text{Ot}}$$ (20o)

$$I_{D}=\{[0.99970]+j[0.03010]\}I_{\text{Ot}}$$ (20p)

$$I_{C_{D}}=\{-[3.0\times 10^{-4}]+j[0.01000]\}I_{\text{Ot}}$$ (20q)

$$I_{\text{Dr}}=\{[0.99940]+j[0.04010]\}I_{\text{Ot}}.$$ (20r)

The 90° out-of-phase parts of shunt currents $I_{C_{5}},I_{C_{3}},I_{C_{1}},I_{C_{D}},\;$ and $I_{C_{A}}$ are again much larger than for shunt currents $I_{C_{2}},I_{C_{4}},I_{C_{S}},\;$ and $I_{C_{B}}$ because contact pads 5*′*, 3*′*, 1*′*, and D*′* are all near the quantum Hall potential, rather than near the shield potential. A 1 % out-of-phase current once again passes through each of the coaxial cable capacitances *C*_5_, *C*_3_, *C*_1_, and *C*_D_ in this example, which is not necessarily a problem if the bridge Drive can provide this extra 4 % of out-of-phase current to *I*_Dr_.

Expressing Eq. (19a) in the form of Eq. (19b), we find that

$$V_{H}(5,6)=\{1+[2.0\times 10^{-8}]-j[2{.0\times 10}^{-6}]\}R_{H}I_{\text{Ot}},$$ (21a)

$$\Delta_{56}=\{[2.0\times 10^{-8}]-j[2{.0\times 10}^{-6}]\}$$ (21b)

for 100 pF lead capacitances and

$$\Delta_{56}=\{[5.0\times 10^{-8}]-j[5{.0\times 10}^{-6}]\}$$ (22)

for 250 pF coaxial leads.

The 2 × 10^−8^ in-phase correction to *R*_H_ for 100 pF leads is larger than our desired 1 × 10^−8^ *R*_H_ total uncertainty, but a correction could be made to the measurements via the approximate equation

$$\Delta_{56}\approx \{[\omega^{2}C_{S}L_{S}+\omega^{2}C_{5}L_{5}]-j[\omega C_{S}r_{S}+\omega C_{5}r_{5}]\}$$ (23)

that might provide sufficient accuracy. We will therefore consider the single-series “offset” circuit as a possible ac QHRS in a future paper which includes the effects of wire-to-wire capacitances. The approximate equations for the currents will be given in that paper.

## 7. Analysis of the Double-Series Circuit

**Fig. 3:**
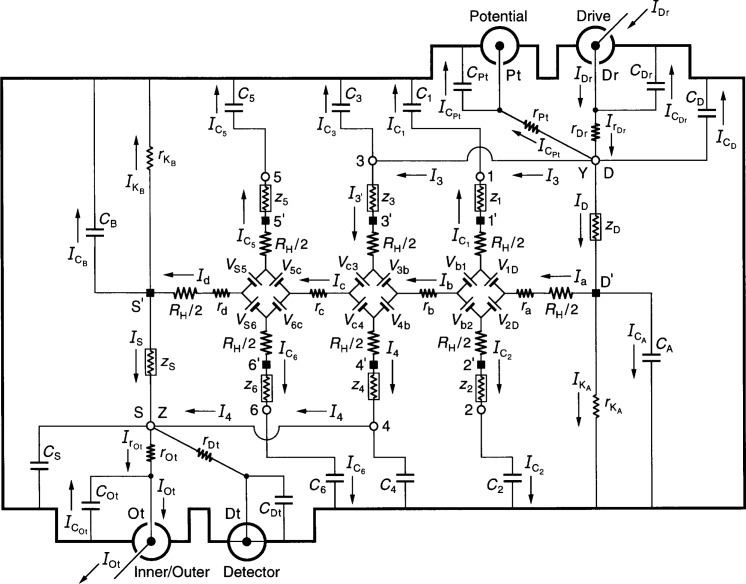
An equivalent electrical circuit representation of an ac QHE resistance standard with two double-series connections to the device. See Sec. 7 for the circuit analysis.

Figure 3 shows an equivalent electrical circuit representation of an ac QHRS using two double-series connections to the QHE device. It is called double-series because there are two current paths to the device provided by a short coaxial lead outside the sample probe that connects room temperature access points 3 and D at point Y. Another short coaxial lead connects access points 4 and S at point Z. Short coaxial leads connect point Y with the Drive and Potential ports, and point Z with the Inner/Outer and Detector ports. For simplicity, we have placed all the parasitic impedances of the short coaxial cables in the cables and coaxial connectors labeled Ot, Dt, Pt, and Dr. These connections were first used by Delahaye [24] in ac quantized Hall resistance measurements (but points Y and Z were at the sample holder rather than outside the cryostat). Most subsequent ac experiments have used double-series or triple-series connections.

### 7.1 Exact Double-Series Equations

To simplify the final algebraic expressions, we again define substitutions of variables, and substitutions of substitutions. Let

$$A=j\omega C_{\text{Ot}}r_{\text{Ot}}$$ (24a)

$$B=j\omega C_{B}z_{S}$$ (24b)

$$C=\frac{1}{j\omega C_{B}r_{K_{B}}}$$ (24c)

$$D=\frac{1}{\left[1+j\omega C_{6}(R_{H}+z_{6})\right]}\frac{C_{6}}{C_{B}}$$ (24d)

$$E=\frac{j\omega C_{6}r_{d}}{\left[1+j\omega C_{6}(R_{H}+z_{6})\right]}$$ (24e)

$$F=\frac{1}{\left[1+j\omega C_{5}z_{5}\right]}\frac{C_{5}}{C_{B}}$$ (24f)

$$G=\frac{j\omega C_{5}(R_{H}+r_{d})}{\left[1+j\omega C_{5}z_{5}\right]}$$ (24g)

$$H_{1}=\frac{z_{S}}{\left(R_{H}+z_{4}\right)}$$ (24h)

$$H_{2}=\frac{r_{d}}{\left(R_{H}+z_{4}\right)}$$ (24i)

$$H_{3}=\frac{r_{c}}{\left(R_{H}+z_{4}\right)}$$ (24j)

$$H_{4}=\frac{R_{H}}{\left(R_{H}+z_{4}\right)}$$ (24k)

$$I=H_{2}+H_{3}(1+G)+E(H_{3}-H_{4})$$ (24l)

$$J=I(1+C)+FH_{3}+D(H_{3}-H_{4})$$ (24m)

$$K=\frac{1}{\left[1+H_{1}+I+BJ\right]}$$ (24n)

$$L=j\omega C_{3}r_{c}$$ (24o)

$$M_{1}=\frac{C_{3}}{C_{5}}+j\omega C_{3}(R_{H}+z_{5})$$ (24p)

$$M_{2}=j\omega C_{3}z_{3}$$ (24q)

$$N_{1}=\frac{j\omega C_{2}r_{b}}{\left[1+j\omega C_{2}(R_{H}+z_{2})\right]}$$ (24r)

$$N_{2}=\frac{j\omega C_{2}z_{4}}{\left[1+j\omega C_{2}(R_{H}+z_{2})\right]}$$ (24s)

$$O_{1}=\frac{j\omega C_{1}r_{b}}{\left[1+j\omega C_{1}z_{1}\right]}$$ (24t)

$$O_{2}=\frac{j\omega C_{1}(R_{H}+z_{3})}{\left[1+j\omega C_{1}z_{1}\right]}$$ (24u)

$$O_{3}=\frac{C_{1}}{C_{3}[1+j\omega C_{1}z_{1}]}$$ (24v)

$$P_{1}=\frac{\left(R_{H}+r_{a}\right)}{r_{K_{A}}}$$ (24w)

$$P_{2}=\frac{z_{2}}{r_{K_{A}}}+\frac{1}{j\omega C_{2}r_{K_{A}}}$$ (24x)

$$Q_{1}=j\omega C_{A}r_{K_{A}}$$ (24y)

$$Q_{2}=r_{a}{+z}_{D}[1+P_{1}(1+Q_{1})]$$ (24z)

$$Q_{3}=z_{D}P_{2}(1+Q_{1})$$ (25a)

$$Q_{4}=r_{b}+Q_{2}+O_{1}(R_{H}+Q)$$ (25b)

$$Q_{5}=Q_{4}+N_{1}(Q_{2}+Q_{3})$$ (25c)

$$Q_{6}=R_{H}+z_{3}+Q_{5}+O_{2}(R_{H}+Q_{2})-M_{2}O_{3}(R_{H}+Q_{2})$$ (25d)

$$Q_{7}=\frac{\left[Q_{5}+LO_{3}(R_{H}+Q_{2})\right]}{Q_{6}}$$ (25e)

$$Q_{8}=\frac{\left[Q_{5}+N_{2}(Q_{2}+Q_{3})\right]}{Q_{6}}$$ (25f)

$$Q_{9}=\frac{M_{1}O_{3}(R_{H}+Q_{2})}{Q_{6}}$$ (25g)

$$R=j\omega C_{D}z_{D}$$ (25h)

$$S=\frac{C_{D}}{C_{A}}$$ (25i)

$$T=\frac{C_{\text{Pt}}}{C_{D}}\frac{1}{\left[1+j\omega C_{\text{Pt}}r_{\text{Pt}}\right]}$$ (25j)

$$U_{1}=\frac{C_{\text{Dr}}}{C_{D}}$$ (25k)

$$U_{2}=j\omega C_{\text{Dr}}r_{\text{Dr}}.$$ (25l)

Six of the current solutions are trivial because of the four-terminal-pair definition [15,16]

$$I_{\text{Dt}}=I_{\text{Pt}}=I_{C_{S}}=I_{C_{\text{Dt}}}=I_{C_{4}}=I_{r\text{Dt}}=0.$$ (26a)

The remaining exact equations for the double-series circuit currents are

$$I_{C_{\text{Ot}}}=\frac{A}{\left(1+A\right)}I_{\text{Ot}}$$ (26b)

$$I_{r_{\text{Ot}}}=I_{\text{Ot}}-I_{C_{\text{Ot}}}$$ (26c)

$$I_{S}=KI_{r_{\text{Ot}}}$$ (26d)

$$I_{4}=I_{r_{\text{Ot}}}-I_{S}$$ (26e)

$$I_{C_{B}}=BI_{S}$$ (26f)

$$I_{K_{B}}=CI_{C_{B}}$$ (26g)

$$I_{d}=I_{S}+I_{C_{B}}+I_{K_{B}}$$ (26h)

$$I_{C_{6}}=DI_{C_{B}}+EI_{d}$$ (26i)

$$I_{C_{5}}=FI_{C_{B}}+GI_{d}$$ (26j)

$$I_{c}=I_{d}+I_{C_{5}}+I_{C_{6}}$$ (26k)

$$I_{3\prime }=Q_{7}I_{c}+Q_{8}I_{4}+Q_{9}I_{C_{5}}$$ (26l)

$$I_{C_{3}}=LI_{c}+M_{1}I_{C_{5}}+M_{2}I_{3\prime }$$ (26m)

$$I_{3}=I_{3\prime }+I_{C_{3}}$$ (26n)

$$I_{b}=I_{c}+I_{4}-I_{3\prime }$$ (26o)

$$I_{C_{2}}=N_{1}I_{b}+N_{2}I_{4}$$ (26p)

$$I_{C_{1}}=O_{1}I_{b}-O_{2}I_{3\prime }+O_{3}I_{C_{3}}$$ (26q)

$$I_{a}=I_{b}+I_{C_{1}}+I_{C_{2}}$$ (26r)

$$I_{K_{A}}=P_{1}I_{a}+P_{2}I_{C_{2}}$$ (26s)

$$I_{C_{A}}=Q_{1}I_{K_{A}}$$ (26t)

$$I_{D}=I_{a}+I_{K_{A}}+I_{C_{A}}$$ (26u)

$$I_{C_{D}}=SI_{C_{A}}+RI_{D}$$ (26v)

$$I_{C_{\text{Pt}}}=TI_{C_{D}}$$ (26w)

$$I_{r_{\text{Dr}}}=I_{D}+I_{3}+I_{C_{D}}+I_{C_{\text{Pt}}}$$ (26x)

$$I_{C_{\text{Dr}}}=U_{1}I_{C_{D}}+U_{2}I_{r_{\text{Dr}}}$$ (26y)

$$I_{\text{Dr}}=I_{r_{\text{Dr}}}+I_{C_{\text{Dr}}}.$$ (26z)

The exact equation for the quantum Hall voltage is obtained by summing the voltages between the inner conductors of the Detector coaxial port and the Potential coaxial port. Taking the path through point Z, arm 4, voltage generators *V*_c4_ and *V*_c3_, arm 3, and point Y we find that

$$V_{H}(Y,Z)+R_{H}I_{c}+(R_{H}+z_{4})I_{4}+z_{3}I_{3\prime }-r_{\text{Pt}}I_{C_{\text{Pt}}}$$ (27a)

which can also be expressed as

$$V_{H}(Y,Z)=[1+\Delta_{\text{YZ}}]R_{H}I_{\text{Ot}}.$$ (27b)

### 7.2 A Numerical Example

We investigate the parasitic impedance contributions of the ac QHRS on the measured value of *V*_H_(Y,Z) for a particular example of the double-series circuit by using the cardinal numbers listed in Eqs. (7), plus the following cardinal numbers for the additional circuit elements

$$r_{\text{Ot}}=r_{\text{Dt}}=r_{\text{Pt}}=r_{\text{Dr}}=10^{-3}\Omega$$ (28a)

$$C_{\text{Ot}}=C_{\text{Dt}}=C_{\text{Pt}}=C_{\text{Dr}}=10^{-12}F.$$ (28b)

The numerical results for the currents in Eqs. (26) are

$$I_{C_{\text{Ot}}}=\{[1.0\times 10^{-22}]+j[1.0\times 10^{-11}]\}I_{\text{Ot}}$$ (29a)

$$I_{r_{\text{Ot}}}=\{[1.00000]-j[1.0\times 10^{-11}]\}I_{\text{Ot}}$$ (29b)

$$I_{S}=\{[0.99990]+j[9.0\times 10^{-11}]\}I_{\text{Ot}}$$ (29c)

$$I_{C_{B}}=\{-[1.0\times 10^{-10}]+j[1.0\times 10^{-8}]\}I_{\text{Ot}}$$ (29d)

$$I_{K_{B}}=\{[1.0\times 10^{-12}]+j[1.0\times 10^{-14}]\}I_{\text{Ot}}$$ (29e)

$$I_{d}=\{[0.99990]+j[1.0\times 10^{-8}]\}I_{\text{Ot}}$$ (29f)

$$I_{C_{6}}=\{[1.1\times 10^{-11}]+j[1.0\times 10^{-6}]\}I_{\text{Ot}}$$ (29g)

$$I_{C_{5}}=\{-[1.0\times 10^{-10}]+j[0.01000]\}I_{\text{Ot}}$$ (29h)

$$I_{c}=\{[0.99990]+j[0.01000]\}I_{\text{Ot}}$$ (29i)

$$I_{4}=\{[1.0\times 10^{-4}]-j[1.0\times 10^{-10}]\}I_{\text{Ot}}$$ (29j)

$$I_{3\prime }=\{[1.0\times 10^{-4}]+j[0.01000]\}I_{\text{Ot}}$$ (29k)

$$I_{C_{3}}=\{-[1.0\times 10^{-4}]+j[0.01000]\}I_{\text{Ot}}$$ (29l)

$$I_{3}=\{[1.6\times 10^{-7}]+j[0.02000]\}I_{\text{Ot}}$$ (29m)

$$I_{b}=\{[0.99990]+j[1.0\times 10^{-6}]\}I_{\text{Ot}}$$ (29n)

$$I_{C_{2}}=\{[1.0\times 10^{-11}]+j[1.1\times 10^{-9}]\}I_{\text{Ot}}$$ (29o)

$$I_{C_{1}}=\{-[1.0\times 10^{-12}]+j[0.01000]\}I_{\text{Ot}}$$ (29p)

$$I_{a}=\{[0.99990]+j[0.01000]\}I_{\text{Ot}}$$ (29q)

$$I_{K_{A}}=\{[1.0\times 10^{-8}]+j[1.0\times 10^{-10}]\}I_{\text{Ot}}$$ (29r)

$$I_{C_{A}}=\{-[1.0\times 10^{-6}]+j[1.0\times 10^{-4}]\}I_{\text{Ot}}$$ (29s)

$$I_{D}=\{[0.99990]+j[0.01010]\}I_{\text{Ot}}$$ (29t)

$$I_{C_{D}}=\{-[1.0\times 10^{-4}]+j[0.01000]\}I_{\text{Ot}}$$ (29u)

$$I_{C_{\text{Pt}}}=\{-[1.0\times 10^{-6}]+j[1.0\times 10^{-4}]\}I_{\text{Ot}}$$ (29v)

$$I_{r_{\text{Dr}}}=\{[0.99980]+j[0.04020]\}I_{\text{Ot}}$$ (29w)

$$I_{C_{\text{Dr}}}=\{-[1.0\times 10^{-6}]+j[1.0\times 10^{-4}]\}I_{\text{Ot}}$$ (29x)

$$I_{\text{Dr}}=\{[0.99980]+j[0.04030]\}I_{\text{Ot}}.$$ (29y)

The 90° out-of-phase parts of shunt currents $I_{C_{5}}$, $I_{C_{3}}$, $I_{C_{1}}$, $I_{C_{D}}$, $I_{C_{A}}$, $I_{C_{\text{Pt}}}$, and $I_{C_{\text{Dr}}}$ are again much larger than for shunt currents $I_{C_{2}}$, $I_{C_{6}}$, $I_{C_{B}}$, and $I_{C_{\text{Ot}}}$ because contact pads 5′, 3′, 1′, and D′ are all near the quantum Hall potential, rather than near the shield potential. A 1 % out-of-phase current passes through each of the coaxial cable capacitances *C*_5_, *C*_3_, *C*_1_, and *C*_D_ in this example, which once again is not necessarily a problem if the bridge Drive can provide this extra 4 % of out-of-phase current to *I*_Dr_.

Expressing Eq. (27a) in the form of Eq. (27b), we find that

$$V_{H}(Y,Z)=\{1+[9.8\times 10^{-9}]+j[0.01000]\}R_{H}I_{\text{Ot}},$$ (30a)

$$\Delta_{\text{YZ}}=\{[9.8\times 10^{-9}]+j[0.01000]\}$$ (30b)

for 100 pF lead capacitances and

$$\Delta_{\text{YZ}}=\{[3.2\times 10^{-8}]+j[0.02501]\}$$ (31)

for 250 pF coaxial leads.

The 1 × 10^−8^ *R*_H_ in-phase correction to *R*_H_ for 100 pF leads meets our desired 10^−8^ *R*_H_ absolute accuracy, but there is a 1 % contribution to *V*_H_(Y,Z) in the 90° out-of-phase j term. Auxiliary balances in the NIST high precision ac bridges are not capable of providing out-of-phase adjustment signals larger than 5 × 10^−4^ *R*_H_, so the 1 % out-of-phase signal is unacceptable. The approximate solutions are listed in the next subsection to show the source of this out-of-phase problem.

### 7.3 Approximate Double-Series Solutions

Some of the terms in the following approximate solutions were obtained using the results of the dc double-series analysis of [22]. Most terms were found in a tedious process by changing the individual values of circuit element components by an order of magnitude in the computer program, observing the calculated results, and then finding the algebraic expressions that produced these results. The approximate solutions yield numerical results that agree with the exact numerical results listed in Eqs. (29) and (30) to within at least two significant figures for both the real and imaginary parts, but other terms may need to be added to these approximate equations if the circuit components have values significantly different from those listed in Eqs. (7) and (28).

$$I_{C_{\text{Ot}}}\approx I_{C_{{\text{Ot}}_{a}}}=\{[\omega^{2}C_{\text{Ot}}C_{\text{Ot}}r_{\text{Ot}}r_{\text{Ot}}]+j[\omega C_{\text{Ot}}r_{\text{Ot}}]\}I_{\text{Ot}}$$ (32a)

$$I_{r_{\text{Ot}}}\approx I_{r_{{\text{Ot}}_{a}}}=\{1-j[\omega C_{\text{Ot}}r_{\text{Ot}}]\}I_{\text{Ot}}$$ (32b)

$$\begin{array}{c} I_{S}\approx I_{S_{a}}=\{\left[1-\frac{r_{S}}{R_{H}}\right]+j[\omega C_{6}(r_{S}+r_{d})-\omega C_{5}r_{c}-\omega C_{\text{Ot}}r_{\text{Ot}}]\\ -j\left[\omega \frac{L_{S}}{R_{H}}-\omega \frac{L_{4}}{R_{H}}\frac{r_{S}}{R_{H}}\right]\}I_{\text{Ot}} \end{array}$$ (32c)

$$\begin{array}{c} I_{C_{B}}\approx I_{C_{B_{a}}}=\{-[\omega^{2}C_{B}(C_{6}r_{S}-C_{\text{Ot}}r_{\text{Ot}})r_{S}+\omega^{2}C_{B}L_{S}]\\ +j[\omega C_{B}r_{S}]\}I_{\text{Ot}} \end{array}$$ (32d)

$$I_{K_{B}}\approx I_{K_{B_{a}}}=\left\{\left[\frac{r_{S}}{r_{K_{B}}}\right]+j\left[\omega \frac{L_{S}}{r_{K_{B}}}\right]\right\}I_{\text{Ot}}$$ (32e)

$$I_{d}\approx I_{d_{a}}=\left\{\left[1-\frac{r_{S}}{R_{H}}\right]+j\left[\omega (C_{B}+C_{6})r_{S}-\omega \frac{L_{S}}{R_{H}}\right]\right\}I_{\text{Ot}}$$ (32f)

$$\begin{array}{c} I_{C_{6}}\approx I_{C_{6_{a}}}=\{[\omega^{2}C_{6}C_{6}R_{H}(r_{S}+r_{d})+\omega^{2}C_{6}C_{6}r_{S}r_{6}\\ -\omega^{2}C_{6}L_{S}]+j[\omega C_{6}r_{S}]\}I_{\text{Ot}} \end{array}$$ (32g)

$$\begin{array}{c} I_{C_{5}}\approx I_{C_{5_{a}}}=\{[\omega^{2}C_{5}C_{5}R_{H}r_{5}-\omega^{2}C_{5}C_{6}R_{H}(r_{S}+r_{d})\\ -\omega^{2}C_{B}C_{5}R_{H}(r_{S}+r_{d})]+[\omega^{2}C_{6}C_{6}R_{H}r_{c}]+j[\omega C_{5}R_{H}]\}I_{\text{Ot}} \end{array}$$ (32h)

$$I_{c}\approx I_{c_{a}}=I_{d_{a}}+I_{C_{5_{a}}}+I_{C_{6_{a}}}$$ (32i)

$$I_{4}\approx I_{4_{a}}=\{1-j[\omega C_{\text{Ot}}r_{\text{Ot}}]\}I_{\text{Ot}}-I_{S_{a}}$$ (32j)

$$\begin{array}{c} I_{3\prime }\approx I_{3\prime {\;}_{a}}=\{[\frac{r_{D}}{R_{H}}+\frac{\left(r_{b}+r_{a}\right)}{R_{H}}+\omega^{2}C_{1}(C_{1}-C_{5})R_{H}R_{H}\\ +\omega^{2}C_{1}C_{1}R_{H}(r_{1}-r_{3})]-[\omega^{2}C_{1}C_{6}R_{H}r_{S}+\omega^{2}(C_{A}+C_{5})L_{D}]\\ +j[\omega C_{1}(R_{H}-r_{3})+\omega (C_{A}+C_{5})r_{D}]\}I_{\text{Ot}} \end{array}$$ (32k)

$$\begin{array}{c} I_{C_{3}}\approx I_{C_{3a}}=\{-[\omega^{2}C_{3}C_{5}R_{H}R_{H}+\omega^{2}C_{1}C_{3}R_{H}r_{3}\\ +\omega^{2}C_{3}C_{6}R_{H}r_{S}]+j[\omega C_{3}R_{H}]\}I_{\text{Ot}} \end{array}$$ (32l)

$$I_{3}\approx I_{3_{a}}=I_{3_{a}^{\prime }}+I_{C_{3_{a}}}$$ (32m)

$$I_{b}\approx I_{b_{a}}=I_{c_{a}}+I_{4_{a}}-I_{3_{a}^{\prime }}$$ (32n)

$$\begin{array}{c} I_{C_{2}}\approx I_{C_{2_{a}}}=\{[\omega^{2}C_{2}(C_{2}+C_{6}-C_{1})r_{S}r_{4}+\omega^{2}C_{1}C_{2}R_{H}r_{b}]\\ +\left[\omega^{2}C_{2}(C_{2}-C_{5})R_{H}r_{b}-\omega^{2}C_{2}L_{S}\frac{r_{4}}{R_{H}}\right]\\ +j\left[\omega C_{2}r_{b}+\omega C_{2}\frac{r_{S}r_{4}}{R_{H}}\right]\}I_{\text{Ot}} \end{array}$$ (32o)

$$\begin{array}{c} I_{C_{1}}\approx I_{C_{1_{a}}}=\{\left[\omega^{2}C_{1}(C_{1}-C_{5})R_{H}R_{H}+\omega^{2}C_{1}C_{1}R_{H}(r_{1}-r_{3})\right]\\ +\left[-\omega^{2}C_{1}C_{6}R_{H}r_{S}+\omega^{2}C_{1}L_{D}+\omega^{2}C_{1}L_{1}\frac{r_{S}}{R_{H}}\right]\\ -\left[\omega^{2}(C_{1}+C_{5})L_{S}\frac{r_{S}}{R_{H}}\right]+j[\omega C_{1}R_{H}]\}I_{\text{Ot}} \end{array}$$ (32p)

$$I_{a}\approx I_{a_{a}}=I_{b_{a}}+I_{C_{1_{a}}}+I_{C_{2_{a}}}$$ (32q)

$$I_{K_{A}}\approx I_{K_{A_{a}}}=\left\{\left[\frac{R_{H}}{r_{K_{A}}}\right]+j\left[\omega C_{5}\frac{R_{H}R_{H}}{r_{K_{A}}}\right]\right\}I_{\text{Ot}}$$ (32r)

$$I_{C_{A}}\approx I_{C_{A_{a}}}=\{-[\omega^{2}C_{A}C_{5}R_{H}R_{H}]+j[\omega C_{A}R_{H}]\}I_{\text{Ot}}$$ (32s)

$$I_{D}\approx I_{D_{a}}=I_{a_{a}}+I_{K_{A_{a}}}+I_{C_{A_{a}}}$$ (32t)

$$I_{C_{D}}\approx I_{C_{D_{a}}}=\{-[\omega^{2}C_{D}C_{5}R_{H}R_{H}]+j[\omega C_{D}R_{H}]\}I_{\text{Ot}}$$ (32u)

$$I_{C_{\text{Pt}}}\approx I_{C_{{\text{Pt}}_{a}}}=\{-[\omega^{2}C_{\text{Pt}}C_{5}R_{H}R_{H}]+j[\omega C_{\text{Pt}}R_{H}]\}I_{\text{Ot}}$$ (32v)

$$I_{r_{\text{Dr}}}\approx I_{r_{{\text{Dr}}_{a}}}=I_{D_{a}}+I_{3_{a}}+I_{C_{D_{a}}}+I_{C_{{\text{Pt}}_{a}}}$$ (32w)

$$I_{C_{\text{Dr}}}\approx I_{C_{{\text{Dr}}_{a}}}=\{-[\omega^{2}C_{\text{Dr}}C_{5}R_{H}R_{H}]+j[\omega C_{\text{Dr}}R_{H}]\}I_{\text{Ot}}$$ (32x)

$$I_{\text{Dr}}\approx I_{{\text{Dr}}_{a}}=I_{r_{{\text{Dr}}_{a}}}+I_{C_{{\text{Dr}}_{a}}}.$$ (32y)

As expected, Eqs. (32i) and (32h) suggest that the current $I_{C_{5}}$ in Fig. 3 enters the Drive, goes to point Y, to point D′, through longitudinal resistances *r*_a_, *r*_b_, and *r*_c_, through arm 5, and then exits through capacitance-to-shield *C*_5_. We would have likewise assumed that the current $I_{C_{1}}$ enters the Drive, goes to point Y, to point D′, through *r*_a_, through arm 1, and then exits through *C*_1_. However, the approximate Eqs. (32p) and (32k), where $I_{C_{1}}$ appears in *I*_3′_, suggest that $I_{C_{1}}$ enters the Drive, goes to point Y, to point 3, through arm 3, travels “upstream” through *r*_b_, through arm 1, and then exits through *C*_1_. The current $I_{C_{3}}$, on the other hand, enters the Drive, goes to point Y, to point 3, and then exits through *C*_3_, bypassing the device altogether; this latter effect provides an advantage to double-series connections by reducing shunt currents within the device.

Expressing Eq. (27a) in terms of Eq. (27b), we find that Eq. (33) gives the approximate quantum Hall voltage correction terms.

We see from Eq. (33) that sample probe lead 5, just as in the single-series “normal” case, is the dominant source of the 1 % out-of-phase component of the quantum Hall voltage signal in the numerical example for this double-series connection to the QHE device. The next subsection investigates the effect of removing this lead, which was effective before in the single-series “normal” case of Sec. 5.

$$\begin{array}{c} \Delta_{\text{YZ}}\approx \{\left[\frac{r_{S}r_{4}}{R_{H}R_{H}}+\frac{r_{3}r_{D}}{R_{H}R_{H}}+\frac{r_{3}(r_{b}+r_{a})}{R_{H}R_{H}}+\omega^{2}C_{5}C_{5}R_{H}r_{5}\right]\\ -[\omega^{2}C_{B}C_{5}R_{H}r_{S}+\omega^{2}C_{6}(C_{5}-C_{6})R_{H}r_{S}\\ -\omega^{2}C_{1}(C_{1}-C_{5})R_{H}r_{3}]\\ +[\omega^{2}C_{6}C_{6}R_{H}(r_{d}+r_{c})-\omega^{2}C_{5}(C_{B}+C_{6})R_{H}r_{d}]\\ +[\omega^{2}C_{6}C_{6}r_{S}r_{6}-\omega^{2}C_{1}C_{6}r_{S}r_{3}+\omega^{2}C_{1}C_{1}r_{3}(r_{1}-r_{3})]\\ -\left[\omega^{2}C_{B}L_{S}+\omega^{2}C_{6}(L_{S}+L_{3})+\omega^{2}(C_{A}+C_{5})L_{D}\frac{r_{3}}{R_{H}}\right]\\ +j[\omega C_{5}(R_{H}+r_{d})+\omega C_{1}r_{3}+\omega (C_{B}+C_{6})r_{S}]\\ -j\left[\omega C_{6}(r_{S}+r_{d})+\omega L_{4}\frac{r_{S}}{R_{H}R_{H}}\right]\}. \end{array}$$ (33)

### 7.4 Disconnecting Sample Probe Lead 5

Equation (33) predicts that the out-of-phase term j[*ω C*_5_*R*_H_] in the expression for *Δ*_YZ_ can be reduced by disconnecting coaxial cable 5 at position 5′, where 5′ is either located at the potential contact pad on the QHE device, or at an intermediate contact point in the sample holder. There is a capacitance *C*_5′_ between the QHE device and the shield that replaces capacitance C_5_ in Fig. 3. Also, a shield impedance *z*_5′_ replaces the lead impedance *z*_5_.

If we assume in the numerical examples that

$$C_{5\prime }=C_{A}=C_{B}=1\;\text{pF}$$ (34a)

$$r_{5\prime }=r_{5}=1\;\Omega .$$ (34b)

Then

$$\Delta_{\text{YZ}}=\{[2.0\times 10^{-8}]+j[1.0\times 10^{-4}]\}$$ (35)

when the coaxial lead capacitances are all 100 pF, and

$$\Delta_{\text{YZ}}=\{[9.4\times 10^{-8}]+j[1.1\times 10^{-4}]\}$$ (36)

when they are 250 pF. All experiments which have measured ac values of *V*_H_(Y,Z) for double-series connections have had to remove coaxial lead 5 because of the effects due to the large capacitance-to-shield *C*_5_ presented above.

Equation (33) could be used to apply corrections to the experimental data in order to reduce the 9.4 × 10^−8^ in-phase error in *R*_H_*I*_Ot_. However, our approximate and exact equations do not include the effects of wire-to-wire capacitances; the bridge auxiliary balance could introduce unintentional in-phase contributions because of the large out-of-phase component of *V*_H_(Y,Z); and it is not trivial to measure the value of *C*_5′_ in order to apply the correction with lead 5 disconnected.

### 7.5 Double-Series Connections at the QHE Device

Many experiments have made double-series connections to the QHE device at the bottom of the sample probe by using short bonding wires to form the circuit. Points Y and Z of Fig. 3 are thus moved from outside the sample probe down to the sample holder. There are no coaxial leads connected to points 1, 2, 5, and 6, so their capacitances-to-shield become much smaller. Four coaxial leads labeled Ot, Dt, Pt, and Dr connect the QHE device to the outside world. The double-series circuit shown in Fig. 3 remains exactly the same for this case, as do Eqs. (24) through (27). The values of some circuit components, however, change.

We use the following cardinal values in our numerical example

$$R_{H}=10^{4}\Omega$$ (37a)

$$r_{S}=r_{1}=r_{2}=r_{3}=r_{4}=r_{5}=r_{6}=r_{D}=10^{-3}\;\Omega$$ (37b)

$$r_{a}=r_{b}=r_{c}=r_{d}=10^{-3}\;\Omega$$ (37c)

$$r_{K_{A}}=r_{K_{B}}=10^{12}\;\Omega$$ (37d)

$$r_{\text{Ot}}=r_{\text{Dt}}=r_{\text{Pt}}=r_{\text{Dr}}=1\;\Omega$$ (37e)

$$C_{S}=C_{1}=C_{2}=C_{3}=C_{4}=C_{5}=C_{6}=C_{D}=10^{-12}\;F$$ (37f)

$$C_{A}=C_{B}=10^{-12}\;F$$ (37g)

$$C_{\text{Ot}}=C_{\text{Dt}}=C_{\text{Pt}}=C_{\text{Dr}}=10^{-10}\;F.$$ (37h)

The numerical results for the currents in Eqs. (26) with the double-series leads connected at the bottom of the sample probe are

$$I_{C_{\text{Ot}}}^{B}=\{[1.0\times 10^{-12}]+j[1.0\times 10^{-6}]\}I_{\text{Ot}}$$ (38a)

$$I_{r_{\text{Ot}}}^{B}=\{[1.00000]-j[1.0\times 10^{-6}]\}I_{\text{Ot}}$$ (38b)

$$I_{S}^{B}=\{[1.00000]-j[2.0\times 10^{-6}]\}I_{\text{Ot}}$$ (38c)

$$I_{C_{B}}^{B}=\{-[1.0\times 10^{-10}]+j[1.0\times 10^{-11}]\}I_{\text{Ot}}$$ (38d)

$$I_{K_{B}}^{B}=\{[1.0\times 10^{-15}]+j[1.0\times 10^{-14}]\}I_{\text{Ot}}$$ (38e)

$$I_{d}^{B}=\{[1.00000]-j[2.0\times 10^{-6}]\}I_{\text{Ot}}$$ (38f)

$$I_{C_{6}}^{B}=\{-[1.0\times 10^{-10}]+j[2.0\times 10^{-11}]\}I_{\text{Ot}}$$ (38g)

$$I_{C_{5}}^{B}=\{[1.0\times 10^{-10}]+j[1.0\times 10^{-4}]\}I_{\text{Ot}}$$ (38h)

$$I_{c}^{B}=\{[1.00000]+j[9.8\times 10^{-5}]\}I_{\text{Ot}}$$ (38i)

$$I_{4}^{B}=\{[3.0\times 10^{-7}]+j[1.0\times 10^{-6}]\}I_{\text{Ot}}$$ (38j)

$$I_{3\prime }^{B}=\{[3.0\times 10^{-7}]+j[1.0\times 10^{-4}]\}I_{\text{Ot}}$$ (38k)

$$I_{C_{3}}^{B}=\{-[1.0\times 10^{-8}]+j[1.0\times 10^{-4}]\}I_{\text{Ot}}$$ (38l)

$$I_{3}^{B}=\{[2.9\times 10^{-7}]+j[2.0\times 10^{-4}]\}I_{\text{Ot}}$$ (38m)

$$I_{b}^{B}=\{[1.00000]-j[2.0\times 10^{-6}]\}I_{\text{Ot}}$$ (38n)

$$I_{C_{2}}^{B}=\{[9.8\times 10^{-16}]+j[1.0\times 10^{-11}]\}I_{\text{Ot}}$$ (38o)

$$I_{C_{1}}^{B}=\{[2.0\times 10^{-10}]+j[1.0\times 10^{-4}]\}I_{\text{Ot}}$$ (38p)

$$I_{a}^{B}=\{[1.00000]+j[9.8\times 10^{-5}]\}I_{\text{Ot}}$$ (38q)

$$I_{K_{A}}^{B}=\{[1.0\times 10^{-8}]+j[9.8\times 10^{-13}]\}I_{\text{Ot}}$$ (38r)

$$I_{C_{A}}^{B}=\{-[9.8\times 10^{-9}]+j[1.0\times 10^{-4}]\}I_{\text{Ot}}$$ (38s)

$$I_{D}^{B}=\{[1.00000]+j[2.0\times 10^{-4}]\}I_{\text{Ot}}$$ (38t)

$$I_{C_{D}}^{B}=\{-[9.9\times 10^{-9}]+j[1.0\times 10^{-4}]\}I_{\text{Ot}}$$ (38u)

$$I_{C_{\text{Pt}}}^{B}=\{-[9.8\times 10^{-7}]+j[0.01000]\}I_{\text{Ot}}$$ (38v)

$$I_{r_{\text{Dr}}}^{B}=\{[1.00000]+j[0.01050]\}I_{\text{Ot}}$$ (38w)

$$I_{C_{\text{Dr}}}^{B}=\{-[1.0\times 10^{-6}]+j[0.01000]\}I_{\text{Ot}}$$ (38x)

$$I_{\text{Dr}}^{B}=\{[1.00000]+j[0.02050]\}I_{\text{Ot}}.$$ (38y)

Expressing Eq. (27a) in the form of Eq. (27b), we find that

$$V_{H}^{B}(Y,Z)=\{1-[1.0\times 10^{-10}]+j[9.8\times 10^{-5}]\}R_{H}I_{\text{Ot}},$$ (39a)

$$\Delta_{\mathrm{YZ}}^{B}=\{-[1.0\times 10^{-10}]+j[9.8\times 10^{-5}]\}$$ (39b)

for 100 pF lead capacitances and

$$\Delta_{\text{YZ}}^{B}=\{[1.8\times 10^{-10}]+j[9.5\times 10^{-5}]\}$$ (40)

for 250 pF coaxial leads.

Equation (40) implies a very small in-phase error in *R*_H_*I*_Ot_. This is not supported by measurements, which have observed errors in *R*_H_*I*_Ot_ of order 10^−7^. The discrepancy could either be due to unintentional in-phase contributions from the bridge auxiliary balances arising from the large out-of-phase component of *V*_H_(Y,Z), or because our equations do not include the effects of wire-to-wire capacitances.

To assist laboratories who are making double-series connection measurements at the QHE device we list the additional terms that should be added to the approximate current and quantum Hall voltage solutions given by Eqs. (32) and (33). Those equations which require additional terms are:

$$I_{d}^{B}\approx I_{d_{a}}^{B}=I_{d_{a}}-\{j[\omega C_{\text{Ot}}r_{\text{Ot}}]\}I_{\text{Ot}}$$ (41a)

$$I_{C_{6}}^{B}\approx I_{C_{6_{a}}}^{B}=I_{C_{6_{a}}}+\{j[\omega C_{6}r_{d}]\}I_{\text{Ot}}$$ (41b)

$$I_{C_{5}}^{B}\approx I_{C_{5_{a}}}^{B}=I_{C_{5_{a}}}+\{[\omega^{2}C_{\text{Ot}}C_{5}R_{H}r_{\text{Ot}}]\}I_{\text{Ot}}$$ (41c)

$$I_{4}^{B}\approx I_{4_{a}}^{B}=I_{4_{a}}+\left\{\left[\frac{\left(r_{d}+r_{c}\right)}{R_{H}}\right]\right\}I_{\text{Ot}}$$ (41d)

$$I_{3\prime }^{B}\approx I_{3_{a}^{\prime }}^{B}=I_{3_{a}^{\prime }}+\left\{j\left[\omega \frac{L_{D}}{R_{H}}\right]\right\}I_{\text{Ot}}$$ (41e)

$$I_{K_{A}}^{B}\approx I_{K_{A_{a}}}^{B}=I_{K_{A_{a}}}-\left\{j\left[\omega C_{\text{Ot}}\frac{R_{H}}{r_{K_{A}}}r_{\text{Ot}}+\omega \frac{L_{D}}{r_{K_{A}}}\right]\right\}I_{\text{Ot}}$$ (41f)

$$I_{C_{A}}^{B}\approx I_{C_{A_{a}}}^{B}=I_{C_{A_{a}}}+\{[\omega^{2}C_{\text{Ot}}C_{A}R_{H}r_{\text{Ot}}+\omega^{2}C_{A}L_{D}]\}I_{\text{Ot}}$$ (41g)

$$I_{C_{D}}^{B}\approx I_{C_{D_{a}}}^{B}=I_{C_{D_{a}}}+\{[\omega^{2}C_{\text{Ot}}C_{D}R_{H}r_{\text{Ot}}]\}I_{\text{Ot}}$$ (41h)

$$\begin{array}{c} I_{C_{\text{Pt}}}^{B}\approx I_{C_{P_{\text{ta}}}}^{B}=I_{C_{P_{\text{ta}}}}\\ +\{[\omega^{2}C_{\text{Ot}}C_{\text{Pt}}R_{H}r_{\text{Ot}}+\omega^{2}C_{\text{Pt}}C_{\text{Pt}}R_{H}r_{\text{Pt}}]\}I_{\text{Ot}} \end{array}$$ (41i)

and

$$\begin{array}{c} \Delta_{H}^{B}\approx \Delta_{H}+\{[\omega^{2}C_{5}C_{\text{Ot}}R_{H}r_{\text{Ot}}+\omega^{2}C_{5}C_{\text{Pt}}R_{H}r_{\text{Pt}}\\ -\omega^{2}C_{\text{Ot}}C_{\text{Ot}}r_{\text{Ot}}r_{\text{Ot}}]-[\omega^{2}C_{\text{Pt}}C_{\text{Pt}}r_{\text{Pt}}r_{\text{Pt}}]\\ -j[\omega C_{\text{Ot}}r_{\text{Ot}}+\omega C_{\text{Pt}}r_{\text{Pt}}]\}. \end{array}$$ (42)

We once again caution the reader that these approximate equations do not include the effects of wire-to-wire capacitances. This circuit is not a good candidate for further analysis because the quantized Hall and longitudinal resistances could not be measured on the same cool-down.

## 8. Triple-Series Circuit

The double-series circuit of Fig. 3 could be converted to a triple-series circuit by adding short coaxial leads between points Y and 1 and Z and 6. We do not consider this triple-series circuit since it would involve several additional months of effort to perform the analysis, and the problems found in double-series circuits in Sec. 7 due to large shunt currents through *C*_5_ also occur in triple-series circuits. Either coaxial lead 5 would have to be disconnected at position 5′ at the QHE device end of the sample probe, or the triple-series connections would have to be made at the device. Neither choice satisfies our goal of measuring the ac and dc quantized Hall and longitudinal resistances on the same cool-down. We therefore proceed to quadruple-series connections, which turns out to satisfy our requirements at this stage of analysis.

## 9. Analysis of the Quadruple-Series Circuit

**Fig. 4:**
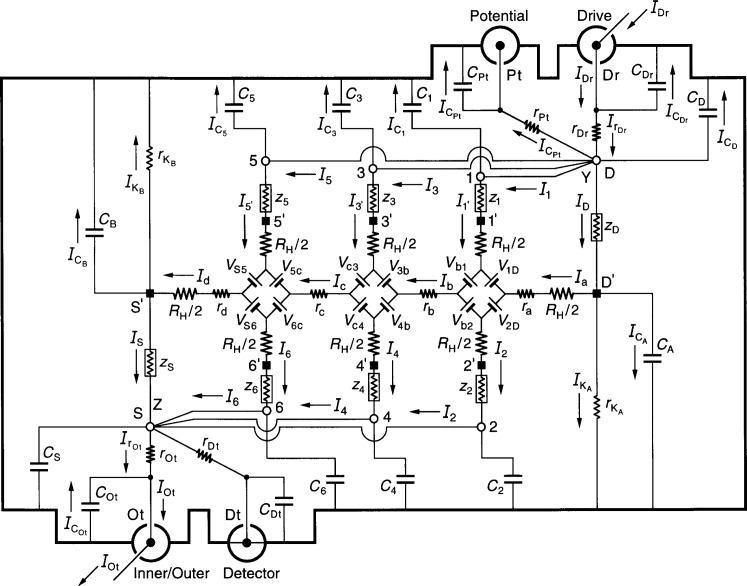
An equivalent electrical circuit representation of an ac QHE resistance standard with two quadruple-series connections to the device. See Sec. 9 for the circuit analysis.

Figure 4 shows an equivalent electrical circuit representation of an ac QHRS using two quadruple-series connections to the QHE device. It is quadruple-series because short coaxial leads outside the sample probe connect room temperature access points 5, 3, 1, and D at point Y, providing four current paths to the device. Other short coaxial leads connect access points 2, 4, 6, and S at point Z. Short coaxial leads outside the sample probe connect point Y with the Drive and Potential ports, and point Z with the Inner/Outer and Detector ports.

### 9.1 Exact Quadruple-Series Equations

To simplify the final algebraic expressions, we once again define substitutions of variables, and substitutions of substitutions. Let

$$A=j\omega C_{\text{Ot}}r_{\text{Ot}}$$ (43a)

$$B=j\omega C_{B}z_{S}$$ (43b)

$$C=\frac{1}{j\omega C_{B}r_{K_{B}}}$$ (43c)

$$D_{1}=\frac{z_{S}}{\left(R_{H}+z_{6}\right)}$$ (43d)

$$D_{2}=\frac{r_{d}}{\left(R_{H}+z_{6}\right)}$$ (43e)

$$D_{3}=\frac{\left(R_{H}+r_{d}\right)}{z_{5}}$$ (43f)

$$D_{4}=\frac{1}{j\omega C_{B}z_{5}}$$ (43g)

$$D_{5}=\frac{1}{j\omega C_{5}z_{5}}$$ (43h)

$$D_{6}=\frac{z_{6}}{\left(R_{H}+z_{4}\right)}$$ (43i)

$$D_{7}=\frac{r_{c}}{\left(R_{H}+z_{4}\right)}$$ (43j)

$$D_{8}=\frac{C_{3}}{C_{5}}$$ (43k)

$$E_{1}=\frac{z_{4}}{\left(R_{H}+z_{2}\right)}$$ (43l)

$$E_{2}=\frac{r_{b}}{\left(R_{H}+z_{2}\right)}$$ (43m)

$$E_{3}=\frac{C_{1}}{C_{3}}$$ (43n)

$$E_{4}=\frac{\left(R_{H}+r_{a}\right)}{r_{K_{A}}}$$ (43o)

$$E_{5}=\frac{z_{2}}{r_{K_{A}}}$$ (43p)

$$E_{6}=j\omega C_{A}r_{K_{A}}$$ (43q)

$$F_{1}=\frac{\left(R_{H}+z_{5}\right)}{z_{3}}$$ (43r)

$$F_{2}=\frac{r_{c}}{z_{3}}$$ (43s)

$$F_{3}=\frac{\left(R_{H}+z_{3}\right)}{z_{1}}$$ (43t)

$$F_{4}=\frac{r_{b}}{z_{1}}$$ (43u)

$$F_{5}=\frac{\left(R_{H}+z_{1}\right)}{z_{D}}$$ (43v)

$$F_{6}=\frac{r_{a}}{z_{D}}$$ (43w)

$$F_{7}=\frac{C_{5}}{C_{A}}$$ (43x)

$$F_{8}=j\omega C_{5}z_{D}$$ (43y)

$$G_{1}=F_{8}+E_{6}(F_{7}+F_{8})$$ (43z)

$$G_{2}=F_{8}+G_{1}(E_{4}+E_{5})$$ (44a)

$$G_{3}=F_{8}+E_{4}G_{1}$$ (44b)

$$G_{4}=G_{3}(1+F_{4})+E_{2}G_{2}$$ (44c)

$$G_{5}=G_{4}+F_{3}G_{3}$$ (44d)

$$G_{6}=G_{4}+E_{1}G_{2}$$ (44e)

$$G_{7}=G_{4}+F_{2}G_{5}+D_{7}G_{6}$$ (44f)

$$G_{8}=G_{7}+D_{6}G_{6}$$ (44g)

$$G_{9}=G_{7}+F_{1}G_{5}$$ (44h)

$$H_{1}=\frac{\left(D_{5}G_{7}-D_{3}\right)}{\left(1+D_{5}G_{9}\right)}$$ (44i)

$$H_{2}=\frac{D_{4}}{\left(1+D_{5}G_{9}\right)}$$ (44j)

$$H_{3}=\frac{D_{5}G_{8}}{\left(1+D_{5}G_{9}\right)}$$ (44k)

$$J_{1}=1+E_{1}+E_{2}$$ (44l)

$$J_{2}=E_{2}(1+F_{2})$$ (44m)

$$J_{3}=J_{2}+D_{7}J_{1}$$ (44n)

$$J_{4}=1+J_{3}+D_{6}J_{1}$$ (44o)

$$J_{5}=J_{3}+E_{2}F_{1}$$ (44p)

$$J_{6}=J_{4}-H_{3}J_{5}$$ (44q)

$$J_{7}=J_{3}-H_{1}J_{5}+D_{2}J_{6}$$ (44r)

$$J_{8}=1+J_{7}+D_{1}J_{6}$$ (44s)

$$J_{9}=J_{7}(1+C)+H_{2}J_{5}$$ (44t)

$$J_{10}=\frac{1}{\left(J_{8}+BJ_{9}\right)}$$ (44u)

$$K_{1}=\frac{C_{D}}{C_{A}}$$ (44v)

$$K_{2}=j\omega C_{D}z_{D}$$ (44w)

$$K_{3}=\frac{C_{\text{Pt}}}{C_{D}}\frac{1}{\left[1+j\omega C_{\text{Pt}}r_{\text{Pt}}\right]}$$ (44x)

$$K_{4}=\frac{C_{\text{Dr}}}{C_{D}}$$ (44y)

$$K_{5}=j\omega C_{\text{Dr}}r_{\text{Dr}}.$$ (44z)

Eight of the current solutions are trivial because of the four-terminal-pair definition [15,16]

$$I_{\text{Dt}}=I_{\text{Pt}}=I_{C_{S}}=I_{C_{\text{Dt}}}=I_{C_{6}}=I_{C_{4}}=I_{C_{2}}=I_{r_{\text{Dt}}}=0.$$ (45a)

The remaining exact equations for the quadruple-series circuit currents are

$$I_{C_{\text{Ot}}}=\frac{A}{\left(1+A\right)}I_{\text{Ot}}$$ (45b)

$$I_{r_{\text{Ot}}}=I_{\text{Ot}}-I_{C_{\text{Ot}}}$$ (45c)

$$I_{S}=J_{10}I_{r_{\text{Ot}}}$$ (45d)

$$I_{C_{B}}=BI_{S}$$ (45e)

$$I_{K_{B}}=CI_{C_{B}}$$ (45f)

$$I_{d}=I_{S}+I_{C_{B}}+I_{K_{B}}$$ (45g)

$$I_{6}=D_{1}I_{S}+D_{2}I_{d}$$ (45h)

$$I_{5\prime }=H_{1}I_{d}-H_{2}I_{C_{B}}+H_{3}I_{6}$$ (45i)

$$I_{C_{5}}=G_{7}I_{d}+G_{8}I_{6}-G_{9}I_{5\prime }$$ (45j)

$$I_{5}=I_{5\prime }+I_{C_{5}}$$ (45k)

$$I_{c}=I_{d}-I_{5\prime }+I_{6}$$ (45l)

$$I_{4}=D_{6}I_{6}+D_{7}I_{c}$$ (45m)

$$I_{3\prime }=F_{1}I_{5\prime }-F_{2}I_{c}$$ (45n)

$$I_{C_{3}}=D_{8}I_{C_{5}}$$ (45o)

$$I_{3}=I_{3\prime }+I_{C_{3}}$$ (45p)

$$I_{b}=I_{c}-I_{3\prime }+I_{4}$$ (45q)

$$I_{2}=E_{1}I_{4}-E_{2}I_{b}$$ (45r)

$$I_{1\prime }=F_{3}I_{3\prime }-F_{4}I_{b}$$ (45s)

$$I_{C_{1}}=E_{3}I_{C_{3}}$$ (45t)

$$I_{1}=I_{1\prime }+I_{C_{1}}$$ (45u)

$$I_{a}=I_{b}-I_{1\prime }+I_{2}$$ (45v)

$$I_{K_{A}}=E_{4}I_{a}+E_{5}I_{2}$$ (45w)

$$I_{C_{A}}=E_{6}I_{K_{A}}$$ (45x)

$$I_{D}=I_{a}+I_{K_{A}}+I_{C_{A}}$$ (45y)

$$I_{C_{D}}=K_{1}I_{C_{A}}+K_{2}I_{D}$$ (45z)

$$I_{C_{\text{Pt}}}=K_{3}I_{C_{D}}$$ (46a)

$$I_{r_{\text{Dr}}}=I_{D}+I_{1}+I_{3}+I_{5}+I_{C_{D}}+I_{C_{\text{Pt}}}$$ (46b)

$$I_{C_{\text{Dr}}}=K_{4}I_{C_{D}}+K_{5}I_{r_{\text{Dr}}}$$ (46c)

$$I_{\text{Dr}}=I_{r_{\text{Dr}}}+I_{C_{\text{Dr}}}.$$ (46d)

The exact equation for the quantum Hall voltage is obtained by summing the voltages between the inner conductors of the Detector coaxial port and the Potential coaxial port. Taking the path through point Z, arm 4, voltage generators *V*_c4_ and *V*_c3_, arm 3, and point Y we find that

$$V_{H}(Y,Z)=R_{H}I_{c}+(R_{H}+z_{4})I_{4}+z_{3}I_{3\prime }-r_{\text{Pt}}I_{C_{\text{Pt}}},$$ (47a)

which can also be expressed as

$$V_{H}(Y,Z)=[1+\Delta_{\text{YZ}}]R_{H}I_{\text{Ot}}.$$ (47b)

### 9.2 A Numerical Example

We investigate the parasitic impedance contributions of the ac QHRS on the measured value of *V*_H_(Y,Z) for a particular example of the quadruple-series circuit by using the cardinal numbers listed in Eqs. (7) and Eqs. (28). The numerical results for the currents in Eqs. (45) and (46) are

$$I_{C_{\text{Ot}}}=\{[1.0\times 10^{-22}]+j[1.0\times 10^{-11}]\}I_{\text{Ot}}$$ (48a)

$$I_{r_{\text{Ot}}}=\{[1.00000]-j[1.0\times 10^{-11}]\}I_{\text{Ot}}$$ (48b)

$$I_{S}=\{[0.99990]-j[1.0\times 10^{-6}]\}I_{\text{Ot}}$$ (48c)

$$I_{C_{B}}=\{-[1.0\times 10^{-10}]+j[1.0\times 10^{-8}]\}I_{\text{Ot}}$$ (48d)

$$I_{K_{B}}=\{[1.0\times 10^{-12}]+j[1.0\times 10^{-14}]\}I_{\text{Ot}}$$ (48e)

$$I_{d}=\{[0.99990]-j[9.9\times 10^{-7}]\}I_{\text{Ot}}$$ (48f)

$$I_{6}=\{[1.0\times 10^{-4}]+j[1.0\times 10^{-6}]\}I_{\text{Ot}}$$ (48g)

$$I_{5\prime }=\{[1.0\times 10^{-7}]+j[3.1\times 10^{-14}]\}I_{\text{Ot}}$$ (48h)

$$I_{C_{5}}=\{-[1.0\times 10^{-10}]+j[0.01000]\}I_{\text{Ot}}$$ (48i)

$$I_{5}=\{[1.0\times 10^{-7}]+j[0.01000]\}I_{\text{Ot}}$$ (48j)

$$I_{c}=\{[1.00000]+j[9.8\times 10^{-9}]\}I_{\text{Ot}}$$ (48k)

$$I_{4}=\{[1.1\times 10^{-7}]+j[2.0\times 10^{-10}]\}I_{\text{Ot}}$$ (48l)

$$I_{3\prime }=\{[1.1\times 10^{-7}]+j[2.0\times 10^{-10}]\}I_{\text{Ot}}$$ (48m)

$$I_{C_{3}}=\{-[1.0\times 10^{-10}]+j[0.01000]\}I_{\text{Ot}}$$ (48n)

$$I_{3}=\{[1.1\times 10^{-7}]+j[0.01000]\}I_{\text{Ot}}$$ (48o)

$$I_{b}=\{[1.00000]+j[9.8\times 10^{-9}]\}I_{\text{Ot}}$$ (48p)

$$I_{2}=\{[1.0\times 10^{-7}]+j[3.1\times 10^{-14}]\}I_{\text{Ot}}$$ (48q)

$$I_{1\prime }=\{[1.0\times 10^{-4}]+j[1.0\times 10^{-6}]\}I_{\text{Ot}}$$ (48r)

$$I_{C_{1}}=\{-[1.0\times 10^{-10}]+j[0.01000]\}I_{\text{Ot}}$$ (48s)

$$I_{1}=\{[1.0\times 10^{-4}]+j[0.01000]\}I_{\text{Ot}}$$ (48t)

$$I_{a}=\{[0.99990]-j[1.0\times 10^{-6}]\}I_{\text{Ot}}$$ (48u)

$$I_{K_{A}}=\{[1.0\times 10^{-8}]-j[1.0\times 10^{-14}]\}I_{\text{Ot}}$$ (48v)

$$I_{C_{A}}=\{[1.0\times 10^{-10}]+j[1.0\times 10^{-4}]\}I_{\text{Ot}}$$ (48w)

$$I_{D}=\{[0.99990]+j[9.9\times 10^{-5}]\}I_{\text{Ot}}$$ (48x)

$$I_{C_{D}}=\{-[1.0\times 10^{-10}]+j[0.01000]\}I_{\text{Ot}}$$ (48y)

$$I_{C_{\text{Pt}}}=\{-[1.0\times 10^{-12}]+j[1.0\times 10^{-4}]\}I_{\text{Ot}}$$ (48z)

$$I_{r_{\text{Dr}}}=\{[1.00000]+j[0.04020]\}I_{\text{Ot}}$$ (49a)

$$I_{C_{\text{Dr}}}=\{-[1.4\times 10^{-12}]+j[1.0\times 10^{-4}]\}I_{\text{Ot}}$$ (49b)

$$I_{\text{Dr}}=\{[1.00000]+j[0.04030]\}I_{\text{Ot}}.$$ (49c)

The 90° out-of-phase parts of shunt currents $I_{C_{5}}$, $I_{C_{3}}$, $I_{C_{1}}$, $I_{C_{D}}$, $I_{C_{A}}$, $I_{C_{\text{Pt}}}$, and $I_{C_{\text{Dr}}}$ are much larger than for shunt currents $I_{C_{B}}$ and $I_{C_{\text{Ot}}}$ because contact pads 5′, 3′, 1′, and D′ are all near the quantum Hall potential, rather than near the shield potential. A 1 % out-of-phase current passes through each of the coaxial cable capacitances *C*_5_, *C*_3_, *C*_1_, and *C*_D_ in this numerical example, which is not necessarily a problem if the bridge Drive can provide this extra 4 % of out-of-phase current to *I*_Dr_. We can see from the small out-of-phase components of currents *I*_5′_, *I*_3′_, *I*_1′_, and *I*_D_ that the four shunt currents $I_{C_{5}}$, $I_{C_{3}}$, $I_{C_{1}}$, and $I_{C_{D}}$ all bypass the QHE device, which is a great advantage of the quadruple-series circuit.

Expressing Eq. (47a) in the form of Eq. (47b), we find that

$$V_{H}(Y,Z)=\{1-[2.0\times 10^{-7}]+j[1.0\times 10^{-8}]\}R_{H}I_{\text{Ot}},$$ (50a)

$$\Delta_{\text{YZ}}=\{-[2.0\times 10^{-7}]+j[1.0\times 10^{-8}]\}$$ (50b)

for 100 pF lead capacitances, and also the same value

$$\Delta_{\text{YZ}}=\{-[2.0\times 10^{-7}]+j[1.0\times 10^{-8}]\}$$ (51)

for 250 pF coaxial leads.

There is only a 1 × 10^−8^ *R*_H_*I*_Ot_ out-of-phase component in the *V*_H_(Y,Z) signal for the numerical examples given in Eqs. (50) and (51). Unlike the double-series circuit, this out-of-phase result is very promising. The real part of *V*_H_(Y,Z), however, appears to have a very large error term that is −2 × 10^−7^ *R*_H_*I*_Ot_ in these two examples; but *V*_H_(Y,Z) actually is the quantized Hall voltage *V*_H_ across the device *minus* the longitudinal voltage *V_x_*(2,6) along the device between points 2 and 6,

$$V_{H}(Y,Z)\equiv V_{H}-V_{x}(2,6),$$ (52a)

and

$$V_{x}(2,6)\approx (r_{b}+r_{c})I_{\text{Ot}}.$$ (52b)

Therefore,

$$V_{H}\approx \left[1+\Delta_{H}+\frac{\left(r_{b}+r_{c}\right)}{R_{H}}\right]R_{H}I_{\text{Ot}},$$ (53a)

or

$$V_{H}\approx [1+\delta_{H}]R_{H}I_{\text{Ot}}.$$ (53b)

*V*_H_ has a correction factor *δ*_H_ in the real term that is only −7.9 × 10^−11^ *R*_H_*I*_Ot_ for these two numerical examples where (*r*_b_ + *r*_c_) = 2 × 10^−7^ *R*_H_.

The quadruple-series circuit is an excellent candidate as a possible ac QHRS, and will be further considered in a future paper which includes the effects of wire-to-wire capacitances. The approximate equations for the currents and quantum Hall voltage will be given in that paper. In the meantime, some approximate equations can found in Ref. [23].

## 10. Conclusions

We have used an equivalent electrical circuit model of the quantum Hall effect device to calculate the effects of parasitic impedances that are present in four-terminal-pair [15,16] measurements of ac quantized Hall resistance standards. The discrete circuit components include all of the parasitic capacitances, inductances, and leakage resistances of the standard except the wire-to wire-capacitances.

Exact algebraic equations have been derived for the currents and quantum Hall voltages for single-series “normal”, single-series “offset”, double-series, and quadruple-series circuit connections to the device. We find that the single-series “offset” and quadruple-series connections appear to meet our desired goals of measuring both the quantized Hall resistance *R*_H_ and the longitudinal resistance *R_x_* in the same cool-down for both ac and dc currents with an absolute accuracy of 10^−8^ *R*_H_ or better. These two circuits will be further considered in a future paper in which the effects of wire-to-wire capacitances are also included in the analysis.
